# Towards harmonized holmium-166 SPECT image quality for dosimetry: a multi-center, multi-vendor study

**DOI:** 10.1186/s40658-025-00733-8

**Published:** 2025-03-19

**Authors:** Lovisa E. L. Westlund Gotby, Martina Stella, Camille D. E. Van Speybroeck, Daphne Lobeek, Floris H. P. van Velden, Mette K. Stam, Petra Dibbets-Schneider, Daphne M. V. de Vries-Huizing, Erik-Jan Rijkhorst, Berlinda J. de Wit-van de Veen, Roel Wierts, Rob van Rooij

**Affiliations:** 1 Department of Medical Imaging, Radboud University Medical Center, Geert Grooteplein Zuid 10, 6525 GA Nijmegen, The Netherlands; 2 Department of Radiology and Nuclear Medicine, University Medical Centre Utrecht, Heidelberglaan 100, 3584CX Utrecht, The Netherlands; 3 Department of Radiology, Section of Nuclear Medicine, Leiden University Medical Center, Albinusdreef 2, 2333 ZA Leiden, The Netherlands; 4 Department of Nuclear Medicine, Netherlands Cancer Institute, Plesmanlaan 121, 1066CX Amsterdam, The Netherlands; 5 Department of Radiology and Nuclear Medicine, Maastricht University Medical Center, P. Debyelaan 25, 6299 HX Maastricht, The Netherlands

**Keywords:** Holmium-166, SPECT/CT, Radioembolization, Phantom study, Image quality, Dual-energy window scatter correction, Triple-energy window scatter correction, Monte Carlo-based scatter correction, Dosimetry

## Abstract

**Background:**

Reliable dosimetry based on SPECT/CT imaging is essential to achieve personalized ^166^Ho-radioembolization treatment planning and evaluation. This study quantitatively evaluates multiple acquisition and reconstruction protocols for ^166^Ho-SPECT imaging based on data from five Dutch hospitals. We aim to recommend an imaging protocol which harmonizes ^166^Ho-SPECT images for reproducible and accurate dosimetry in a multi-scanner and multi-center setting.

**Methods:**

Cylindrical and NEMA IEC phantoms, filled with ^166^Ho-chloride, were imaged using seven SPECT/CT scanners from two vendors (GE HealthCare and Siemens Healthineers). Data were acquired with a photopeak window centered at 81 keV. Two adjacent scatter windows, and one upper scatter window at 118 keV were used for triple-energy window (TEW) and dual-energy window (DEW) scatter correction, respectively. The TEW and DEW reconstructions used vendor-specific software. Additionally, a vendor-neutral software package with Monte Carlo (MC) scatter correction (Hermes Medical Solutions) was used to study the influence of scanner hardware on the image quality. System sensitivity was measured in projection data of the cylindrical phantom. The axial uniformity in the cylindrical phantom was used to characterize the impact of the scatter correction method. The image quality was evaluated by the coefficient of variation (COV; noise), the contrast recovery coefficients (CRCs) and contrast-to-noise ratios (CNRs).

**Results:**

TEW scatter correction resulted in superior uniformity and higher CRCs compared to the DEW (CRC for the largest sphere over all scanners, mean ± SD (range): TEW 0.54 ± 0.07 (0.36–0.65), DEW 0.44 ± 0.04 (0.34–0.51)). DEW resulted in lower noise levels compared to TEW (16% lower on average). The DEW and TEW images resulted in comparable CNRs. The system sensitivities and the vendor-neutral image reconstructions demonstrated differences in hardware between the two vendors, most likely due to the characteristics of the vendor-specific medium energy collimator.

**Conclusion:**

This study demonstrates that TEW scatter correction increases the accuracy of ^166^Ho-SPECT images compared to DEW, and we henceforth recommend adopting this method in the clinical ^166^Ho-dosimetry workflow. Scanner hardware has a substantial impact on the characteristics of the acquired data, and identical reconstruction settings will therefore not automatically lead to harmonized image quality.

**Supplementary Information:**

The online version contains supplementary material available at 10.1186/s40658-025-00733-8.

## Background

Accurate personalized dosimetry is an important step to improve the efficacy of radioembolization treatments [1]. In the clinical workflow, dosimetry is utilized during the planning procedure to determine whether a sufficient tumor absorbed dose can be achieved, while verifying whether the healthy tissue can be spared. After the treatment, dosimetry is utilized to evaluate treatment outcome by assessing the tumor and healthy-liver absorbed doses, essential to establish dose–response relationships [2].

Dosimetry for holmium-166 (^166^Ho) microsphere treatments can be carried out based on single photon emission computed tomography/computed tomography (SPECT/CT) images. The accuracy of SPECT-based dosimetry depends on the image quality, which depends on the measured radionuclide’s emission characteristics, acquisition and reconstruction parameters and scanner hardware. In multi-scanner, multi-center studies, it is of particular importance to know the impact of these parameters on the accuracy of the dosimetry. Ideally, standardized protocols for ^166^Ho-SPECT image acquisition and reconstruction should be defined to ensure consistent image quality amongst different scanners. General initiatives for standardization of quantitative SPECT/CT imaging have previously been taken [3], and radionuclide specific initiatives have been taken for technetium-99m, lutetium-177, iodine-123, and iodine-131 [4–7]. For positron emission tomography (PET), an initiative for standardization and harmonization of image quality and quantification commenced in 2006 when the European Association of Nuclear Medicine (EANM) launched EANM Research4Life GmbH (EARL). Since 2010, imaging sites can be accredited when they meet the requirements of the EARL guideline [8], this enhances the comparability of acquired PET data and makes it a standard diagnostic modality in clinical trials. Given the increased use of ^166^Ho as radioembolization device, ^166^Ho-SPECT imaging would also benefit from standardized image quality and a harmonized quantification workflow.

^166^Ho decay results in the emission of a beta particle (max energy/yield: 1.77 MeV/50.5%, 1.85 MeV/48.2%), used for therapy, and relatively low energy gamma radiation, which can be used for SPECT imaging (80.57 keV/6.55%) [9]. High energy gammas (1.38 MeV/0.93%, 1.58 MeV/0.19%, 1.66 MeV/0.12%) are also emitted [9]. When imaging a patient with SPECT, the main photopeak window (centered at 81 keV) contains a combination of primary photopeak photons, Compton scattered photons, Compton down-scattered high energy gammas, bremsstrahlung photons from the beta particles, and characteristic lead x-rays from the collimator. Ideally, when acquiring and reconstructing ^166^Ho-SPECT data, all these interactions should be estimated and accounted for.

To date, the clinically most used ^166^Ho-SPECT imaging protocol samples the main photopeak at 81 keV (15% width) as well as a scatter window centered at 118 keV (12% width) [10, 11]. Dual-energy window-based scatter correction (DEW), with a window weight (k-factor) of 1.05–1.15 [12–14], has been applied to correct for the down-scattered high energy photons. Initially, the 118 keV window, rather than a window adjacent to the photopeak window, was chosen to allow an additional window at 100 keV which was used to simultaneously acquire gadolinium-153 transmission data (a commonly used method for attenuation correction before the introduction of hybrid SPECT/CT scanners) [15]. Moreover, a lower energy scatter window was not applied because of the presence of characteristic x-rays (k-edge) of holmium at 56 keV as well as characteristic x-rays of lead at 75 keV [15], both of which could either contaminate the lower scatter window or the main photopeak itself [10]. Instead of using a lower scatter window, a Monte Carlo (MC) method can be used to estimate and correct for scatter and lead x-rays in the photopeak window [15, 16]. This scatter correction procedure, however, was not widely available on clinical systems. The DEW approach presents multiple challenges. Firstly, it only corrects the image based on scattered high-energy photons, while the scatter from the main photopeak and lead x-rays are not considered, unless the MC scatter correction method is applied. Secondly, it is not trivial to define an appropriate k-factor for the 118 keV scatter window, as it is dependent on patient geometry. Lastly, the 118 keV upper scatter window is not located directly adjacent to the main photopeak window, and it can therefore be challenging to correctly incorporate this in the reconstruction software. To address these difficulties, an alternative scatter correction technique could be the triple-energy window (TEW) method [17]. The TEW protocol is generally easy to implement into the reconstruction software and it also takes both lower and upper scatter into account. Another possibility is a commercially available MC-based scatter correction for which only the main photopeak window is needed to reconstruct the image (HybridRecon Oncology, Hermes Medical Solutions, Sweden).

In this study, multiple acquisition and reconstruction protocols are assessed with the aim of harmonizing ^166^Ho-SPECT imaging for reproducible and accurate dosimetry in a multi-scanner and multi-center setting. The goal of this work is to propose a standardized workflow for ^166^Ho-SPECT imaging suitable for dosimetry, applicable in a clinical scenario. For this purpose, ^166^Ho-SPECT/CT phantom data from a total of seven SPECT/CT scanners from two different vendors, across five Dutch hospitals, were collected. Based on these data the suitability of DEW-, TEW-, and MC-based scatter correction, with respect to the quantitative accuracy of the images, is evaluated.

## Methods

Image quality was assessed by acquiring phantom data on multiple SPECT/CT scanners from two different vendors evaluating uniformity, noise, contrast recovery, and contrast-to-noise ratio. Five medical centers in the Netherlands participated in the study: Leiden University Medical Center (LUMC), Maastricht University Medical Center (MUMC +), The Netherlands Cancer Institute (NKI), Radboud University Medical Center (Radboudumc), and University Medical Center Utrecht (UMCU). In total, seven SPECT/CT scanners from two vendors were included: one Discovery NM/CT 670 Pro and one Discovery NM/CT 870 DR (GE HealthCare, USA), as well as two Symbia Intevo Bold and three Symbia T16 (Siemens Healthineers, Germany); details of the camera models are given in Table 1. Throughout this article the scanners can be differentiated based on their system ID listed in Table 1.

**Table 1:** Main characteristics of SPECT/CT systems used in this study

Vendor		GE HealthCare		Siemens Healthineers				
Imaging center		Leiden University Medical Center (LUMC)	Maastricht University Medical Center (MUMC +)	Netherlands Cancer Institute (NKI)		Radboud University Medical Center (Radboudumc)		University Medical Center Utrecht (UMCU)
System		GE Discovery NM/CT 670 Pro	GE NM/CT 870 DR	Siemens Symbia Intevo Bold	Siemens Symbia T16	Siemens Symbia Intevo Bold	Siemens Symbia T16	Siemens Symbia T16
System ID		GE 670	GE 870	Siemens Intevo 1	Siemens Symbia 1	Siemens Intevo 2	Siemens Symbia 2	Siemens Symbia 3
Year of Installation		2016	2022	2021	2008	2019	2011	2011
Software version		Xeleris 4.0 Acquisition console: 1.003.435.0	Xeleris 4.16 Acquisition console: 1.003.672.8	VB22A	VB10E	VB21A	VB10E	VB10E
SPECT detector	Detector crystal	9.5 mm (3/8 in) NaI		9.5 mm (3/8 in) NaI				
	Field-of-view [cm^2^]	54 × 40		53.3 × 38.7				
Collimator	Name	Medium Energy General Purpose (MEGP)		Medium Energy Low Penetration (MELP)				
	Hole shape	Hex		Hex				
	Hole diameter [mm]	3.0		2.94				
	Septal thickness [mm]	1.05		1.14				
	Hole length [mm]	58		40.64				
	Weight [kg]	103		63.5				

### Phantom preparation

At each imaging center, a cylindrical and a NEMA IEC body phantom were filled with ^166^Ho-chloride (HolmiumSolution, Quirem Medical B.V., The Netherlands [18]) dissolved in water, see Table 2. The activity level was chosen to mimic the scout activity of approximately 300 MBq [19] (QuiremScout™ [20]), while limiting the impact of the dead-time [21, 22]. To ensure homogeneous solutions and prevent sticking (or plating) of activity to the phantom walls, the ^166^Ho-chloride solution was mixed with either ethylenediaminetetraacetic acid or hydrochloric acid according to each imaging center’s own protocol. The cylindrical phantom was homogeneously filled. The NEMA IEC phantom, containing six fillable spheres (inner diameters 10, 13, 17, 22, 28, 37 mm and volumes 0.5, 1.2, 2.6, 5.6, 11.5, 26.5 mL), was filled with a sphere-to-background ratio of approximately 8:1, and equipped with a cold lung insert (outside diameter of 50 mm). Phantom volumes and activity for each center are specified in Table 2.

**Table 2:** Phantoms volumes and activities at scan time for each participating imaging center. All cylindrical phantoms had approximately the same diameter (20–21.5 cm). For the NEMA IEC phantom, the activity is given for the time point of the first scan

Imaging center	Cylinder		NEMA IEC		
	Volume [mL]	Activity [MBq]	Volume background + spheres [mL]	Activity in background + spheres [MBq]	Sphere-to-background ratio
LUMC	6800	269	9756 + 48	261 + 10	8:1
MUMC +	6816	281	9772 + 48	259 + 10	8:1
NKI	9325	293	9883 + 48	289 + 10	7.4:1
Radboudumc	6283	289	9775 + 48	279 + 11	8:1
UMCU	6266	332	9756 + 48	342 + 13	8:1

### Data acquisition

**Fig. 1:**
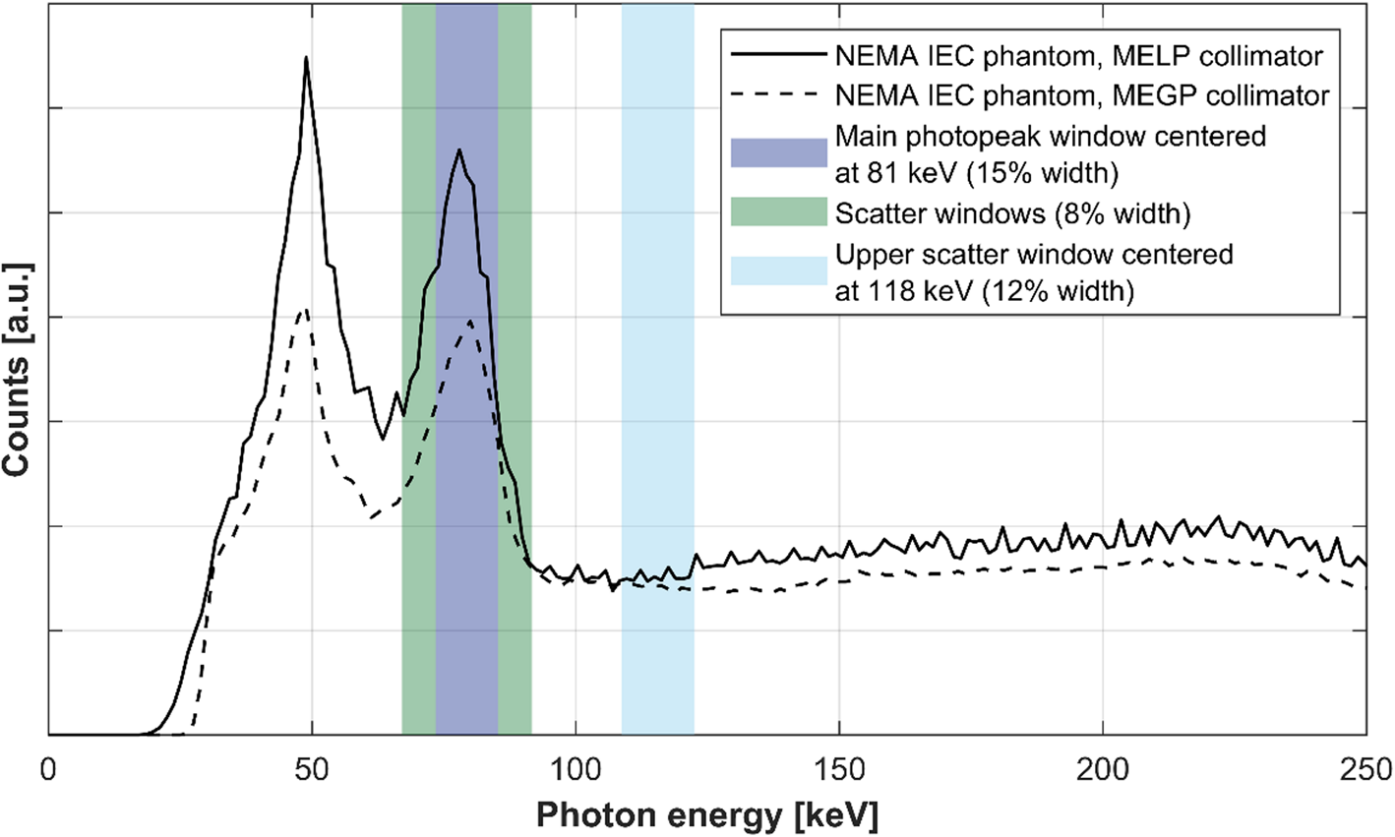
Energy spectra and acquired energy windows. ^166^Ho-spectra for the NEMA IEC phantom acquired with the Siemens MELP (solid black line) and the GE MEGP (dashed black line) collimators are shown. The NEMA spectra were normalized using the data in the upper scatter window, and scaled with the mean measured system sensitivity of all Siemens and GE data in the upper scatter window (Table 3). The main photopeak window is displayed in purple, the adjacent scatter windows are displayed in green (used for the TEW reconstructions), and the upper scatter window is displayed in light blue (used for the DEW reconstructions)

Data were acquired for a photopeak window centered at 81 keV (15% width), for two adjacent scatter windows (8% width), and for one upper scatter window centered at 118 keV (12% width), see Fig. 1. For each scanner, the cylindrical phantom was acquired once, while the NEMA IEC phantom was acquired three times for statistical purposes for all scanners except for GE 670 (logistical issues prevented additional acquisitions). Projections were acquired over a 360° non-circular orbit for a total of 120 projections, 20 s per projection, and a 128 × 128 matrix size. The length of the SPECT acquisition (total time of approximately 20 min) was determined based on the current clinical practice in the participating hospitals. Prior to the acquisitions, each Siemens SPECT/CT scanner had been peaked, as per manufacturer recommendations, using a 5–10 MBq ^166^Ho point source in a syringe (1 mL) positioned at approximately 1 m distance from the detector heads (without collimators) turned to face the syringe. The applied peak shifts were in the range of −2.4 to –1.9%. For GE, SPECT systems are not peaked for individual isotope/energy-window combinations but a dedicated ^166^Ho-uniformity map was created for GE 870. For each SPECT acquisition, a low-dose CT was acquired for attenuation correction and volume of interest (VOI) delineation, using the standard protocol at each imaging center. Additionally, SPECT data with a main photopeak window centered at 81 keV (20% width) and adjacent lower and upper scatter windows (10% width) were acquired to assess the impact of the window width, see supplementary information.

### Data reconstruction

Each SPECT/CT dataset was reconstructed with an ordered subset expectation maximization (OSEM) algorithm with both a vendor-specific (either GE Xeleris or Siemens e.SOFT) and a vendor-neutral software (HybridRecon Oncology, version 4.0.4, Hermes Medical Solutions, Sweden). All reconstructions employed CT-based attenuation correction, and collimator- and detector response-modelling. Reconstructed isotropic voxel size was 4.4 mm and 4.8 mm for data acquired with the GE and Siemens scanners, respectively. Data without post-reconstruction filtering is presented in the main text and post-reconstruction filtered data for the NEMA IEC phantom can be found in the supplementary information. The vendor-neutral software was utilized to elucidate the influence of the scanner hardware on the image quality.

**Fig. 2:**
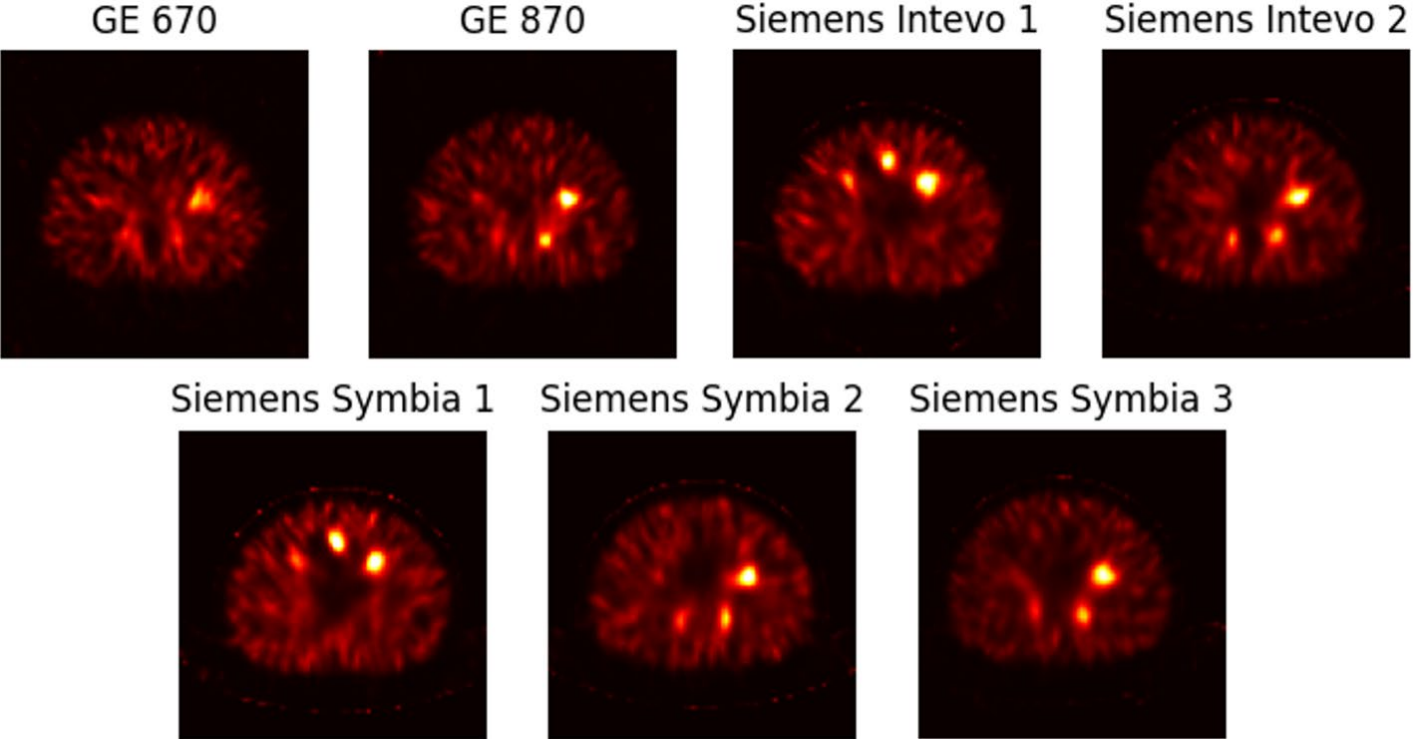
SPECT images of the NEMA IEC phantom, at the plane through the centers of the spheres, for all scanners in this study. The images were reconstructed with vendor-specific software using 4 iterations and 8 subsets for GE, and 10 iterations and 8 subsets for Siemens. No post filtering was applied to the images in this visualization. Note that imaging for GE 670, GE 870, Siemens Intevo 2, Siemens Symbia 2, and Siemens Symbia 3 were acquired with the standard NEMA IEC sphere configuration [23], while another sphere configuration was used for Siemens Intevo 1 and Siemens Symbia 1

As no manufacturer recommendations specific for the reconstruction of ^166^Ho-SPECT images are available, the number of updates used to reconstruct the images was based on either clinical practice or visual assessment of the image quality. For vendor-specific reconstructions, data acquired with Siemens scanners were reconstructed using 10 iterations and 8 subsets (as per clinical practice) [12]. This approach was also evaluated for vendor-specific GE reconstructions of the cylinder data. However, because of the differences in hardware and because of the potential differences in the implementation of the vendor-specific OSEM reconstruction algorithms, and the collimator- and detector response-modelling, the convergence rate and noise build-up can be expected to yield different results. Identical reconstruction parameters in the vendor-specific software do therefore not lead to harmonized image quality. In order for the vendor-specific reconstruction algorithms to produce visually similar images with comparable noise level, the data from the cylindrical phantom and the subsequent NEMA IEC phantom data reconstructed with GE software utilized 4 iterations and 8 subsets, see Fig. 2. The choice of reconstruction parameters for the vendor-specific reconstructions is further elaborated on in the supplementary information. DEW and TEW scatter corrections methods were utilized in the vendor-specific reconstructions. For the DEW scatter correction, the upper scatter energy window centered at 118 keV (12% width) with a k-factor of 1.05 was used. For the TEW scatter correction, the scatter windows adjacent to the photopeak (8% width) were used with automatic window weights of 0.94 to compensate for the window width differences. In the vendor-neutral software, all data were reconstructed with 10 iterations and 8 subsets, with MC-based scatter correction. For the MC-based scatter correction, 100 000 simulated photons, two update iterations for the scatter projections, and 1 000 000 down-scatter photons were used.

### Data analysis

The acquired SPECT projection data were evaluated to determine the sensitivity of each system, and the reconstructed SPECT images were analyzed to assess the impact of the scatter correction method (DEW, TEW or MC) on the uniformity, noise level, contrast recovery and contrast-to-noise ratio. VOI delineation and analysis was carried out using in-house developed scripts in Python (Python Software Foundation. Python Language Reference, version 3.9.12), see Data availability (below) for access to the code.

#### System sensitivity

The system sensitivity for each energy window $i$, in counts per second per megabecquerel [cps/MBq], was determined based on the projection data of the cylindrical phantom measured on each scanner. It was calculated as:

$$system\; sensitivity_{i} = \frac{{counts_{i} }}{t \cdot n \cdot A},$$

where ${counts}_{i}$ is the sum of the counts in all projections (frames) of energy window $i$ [counts], $t$ is the time per projection [s], $n$ is the number of projections, and $A$ is the total activity present in the cylindrical phantom at the start of the scan [MBq].

#### Uniformity in cylindrical phantom

The axial uniformity was measured in the cylindrical phantom for all three different scatter correction methods (DEW, TEW and MC). For each reconstruction, the axial profile was calculated as the sum of reconstructed counts in each slice. The data in each slice was masked with a circular mask dilated 5 voxels outside of the phantom. The shape of the axial profile was considered representative of the effectiveness of the scatter correction method: the ideal profile resembles a rectangular function. Over- or under-correction of the photopeak-scatter gives rise to a central decrease or increase in signal, respectively. A quadratic function of the form $f\left(x\right)={A\frac{x}{L}}^{2}$ was fitted to the normalized and centered axial profile, where L is the phantom length. The coefficient characterizing the curvature of the axial profile, $A$, was used to compare the scatter correction methods; a value of $A$ closer to zero corresponds to a flatter profile, indicating a more accurate scatter correction.

#### Noise

The noise in the reconstructed SPECT images was determined by calculating the coefficient of variation (COV) per slice in a VOI within the cylindrical phantom, and then averaging the COV over the included slices. The COV was defined as $COV=\frac{\sigma }{\mu }$, where $\sigma$ is the population standard deviation (SD) of the counts of the VOI in the slice, and $\mu$ is the mean of the counts in the VOI in the same slice. The VOI was obtained by eroding a CT-based mask of the cylindrical phantom by 30 mm to avoid edge effects.

#### Contrast recovery

**Fig. 3:**
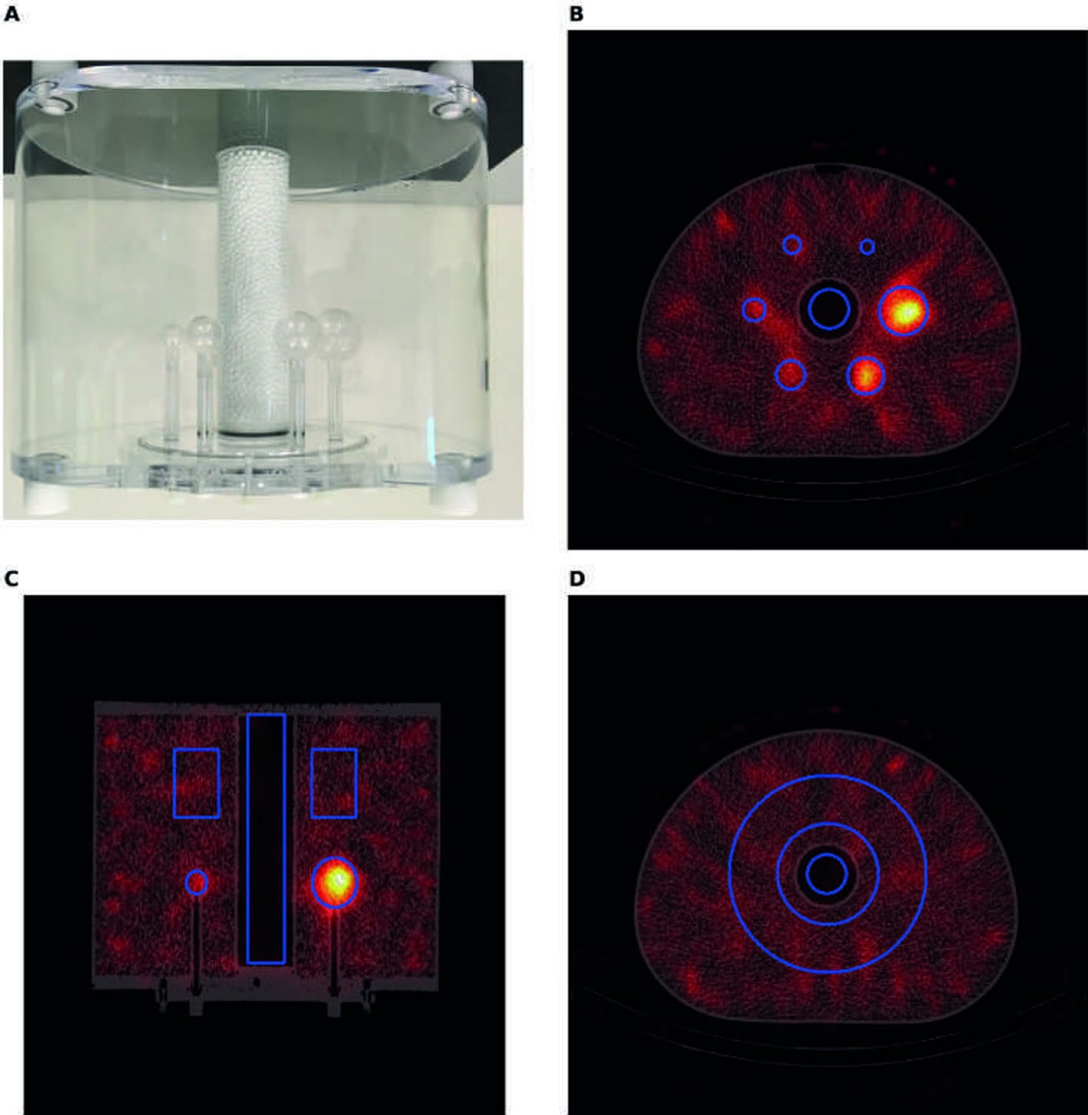
The NEMA IEC body phantom used for the experiment is shown in panel **A**. Panel **B**,** C**, and **D** show the CT fused with the corresponding SPECT acquired at ~ 300 MBq (TEW reconstruction, Siemens Symbia 3) for the axial (plane through the centers of the spheres), coronal (cross-section through lung insert) and axial view (cross-section through background VOI), respectively. VOIs used to assess the contrast recovery coefficients and contrast-to-noise ratios are depicted in blue

For the NEMA IEC phantom assessment, contrast recovery coefficients (CRCs) were computed. The CRCs for the hot spheres and the lung insert were calculated as:

$$CRC = \frac{{\frac{C}{{C_{B} }} - 1}}{R - 1},$$

where $C$ is either ${C}_{S}$, the average number of counts in the sphere VOI, or ${C}_{L}$, the average number of counts in the lung VOI, while ${C}_{B}$ is the average number of counts in the background VOI. R is the ratio between activity concentration in the VOI and in the background, equal to either the nominal sphere-to-background ratio for spheres (see Table 2) or to 0 for the lung, respectively [23]. A CRC of 1 represents perfect recovery of the contrast in the investigated VOI. The six spherical VOIs (true to physical inner diameter) were manually centered on the spheres in a reference CT (for each scanner). For each SPECT image, the accompanying low-dose CT was automatically registered to the reference CT, thereby transforming the VOIs. For the lung, a cylindrical VOI with a diameter of 30 mm, and a height extending along the whole length of the phantom, was centered on the lung insert. The diameter of the lung VOI was chosen in accordance with the NEMA standards [23]. The background VOI was defined as a cylindrical shell, centered around the lung insert, within the uniformly filled background compartment of the NEMA IEC phantom. This cylindrical shell was axially positioned equidistant from the spheres and the edge of the phantom, with a radius of revolution of 57 mm, a thickness of 37 mm (matching the largest sphere), and a height of 50 mm. The shell location was chosen to obtain comparable scatter and attenuation as for the sphere VOIs. All the VOIs were automatically registered based on CT and are shown in Fig. 3.

#### Contrast-to-noise ratio

To assess the overall image quality, i.e. the detectability of hot and cold objects in the phantom, contrast-to-noise ratio (CNR) was measured as:

$$CNR=\frac{C-{C}_{B}}{{\sigma }_{B}}$$

where $C$ and ${C}_{B}$ are defined as above, and ${\sigma }_{B}$ is the SD in the background VOI (cylindrical shell in NEMA IEC phantom, see Fig. 3).

## Results

For the reconstructions of the cylindrical phantom with the vendor-specific software of the GE data (DEW and TEW scatter corrections), 4 iterations and 8 subsets were utilized for the uniformity results, and for the noise (COV), results for both 4 iterations and 8 subsets, as well as for 10 iterations and 8 subsets are presented. All other cylindrical phantom data were reconstructed using 10 iterations and 8 subsets.

For the NEMA IEC phantom reconstructions, the assessed metrics are presented as the average values of the three measurements for each scanner and reconstruction combination, except for GE 670 where only one NEMA dataset was acquired. The results for the additional acquisitions with wider energy window width (main photopeak 20% wide) are reported in the supplementary information and referred to in the text only when relevant.

### System sensitivity

System sensitivities based on the projection data of the cylindrical phantoms, measured as cps/MBq, in each acquired energy window are reported in Table 3. Data acquired with the GE MEGP collimators resulted in approximately 35% lower system sensitivity in the main photopeak window compared to data acquired with the Siemens MELP collimators.

**Table 3:** System sensitivity [cps/MBq] per acquired energy window for each of the SPECT/CT systems included in this study

System ID	Low scatter window [cps/MBq]	Main photopeak window at 81 keV [cps/MBq]	High scatter window [cps/MBq]	Upper scatter window at 118 keV [cps/MBq]
GE 670	2.16	4.54	1.42	2.67
GE 870	2.00	4.50	1.29	2.28
Siemens Intevo 1	2.70	6.64	1.57	2.51
Siemens Intevo 2	2.96	7.04	1.79	2.83
Siemens Symbia 1	2.68	6.53	1.63	2.44
Siemens Symbia 2	2.85	7.03	1.89	2.77
Siemens Symbia 3	3.15	7.72	2.09	3.00

### Uniformity in cylindrical phantom

**Fig. 4:**
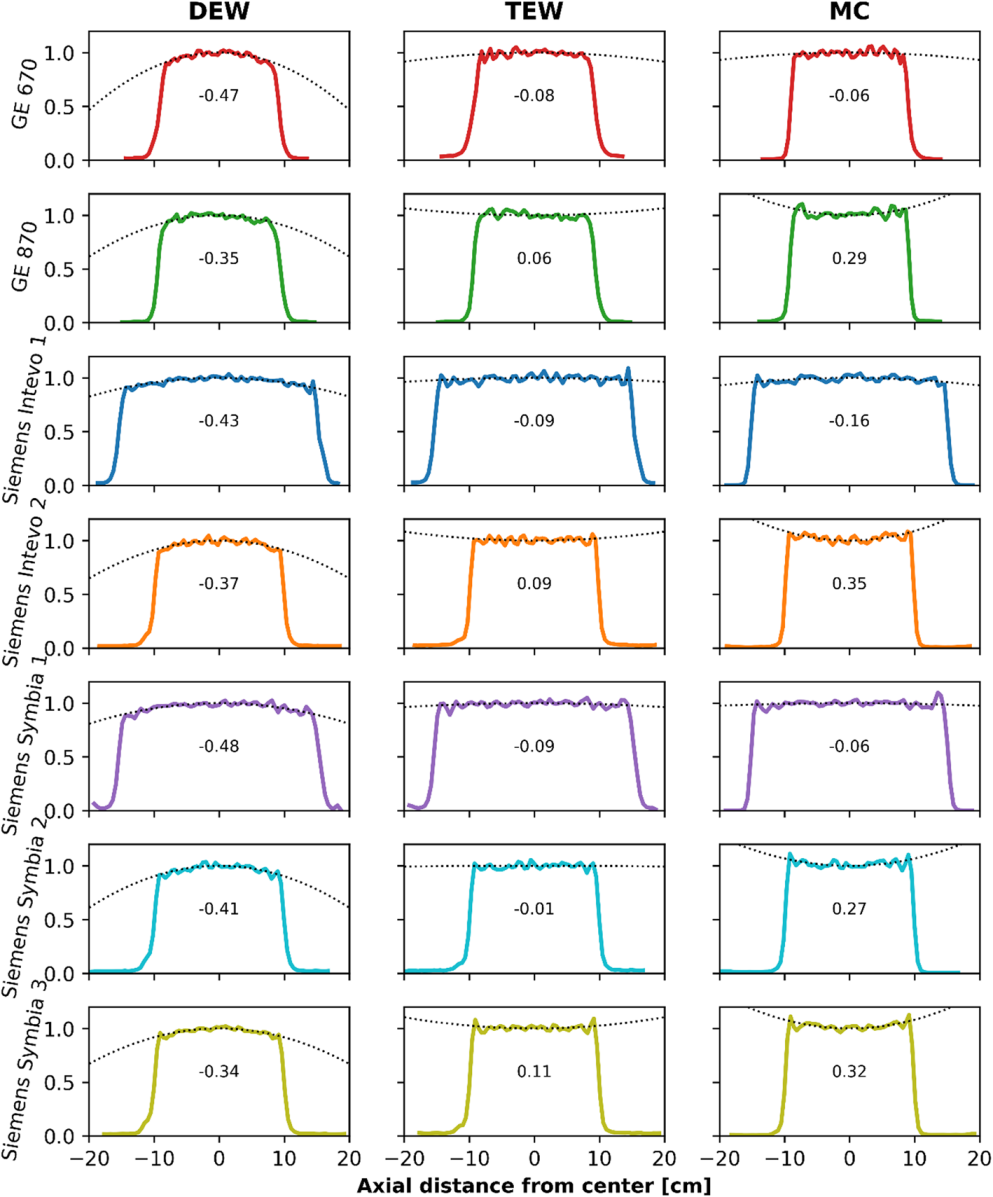
Line profiles measured along the axial direction (along the length) of the cylindrical phantom acquired for each scanner. The dotted lines indicate the fitted curvature along the tops of the line profiles (extended outwards for sake of clarity). For each plot, the curvature coefficient $A$ is reported

Line profiles, representing image uniformity, are displayed in Fig. 4 for each scanner and for each of the three scatter correction methods (DEW, TEW and MC) investigated. The magnitude of the $A$ coefficient was larger for DEW reconstructions than for both TEW and MC reconstructions, when comparing each scanner separately, indicating an increase in counts in the central part of the phantom due to photopeak scatter and hence a less suitable scatter correction. Averaged over all scanners, the DEW reconstructions resulted in an $A$ coefficient of -0.41 ± 0.05 (mean ± SD), the TEW in 0.00 ± 0.08 and MC in 0.14 ± 0.20.

### Noise

Noise, measured as an average COV over the slices within the reconstructions of the cylindrical phantom, is reported in Table 4. For vendor-specific reconstructions, DEW globally yielded lower average COV compared to TEW (16% lower on average). The influence of the hardware (collimator) differences between the GE and the Siemens scanners becomes apparent with the vendor-neutral reconstruction algorithm (MC; all data reconstructed with identical settings), as the COV ranges from 0.17 to 0.38.

**Table 4:** Noise, measured as the coefficient of variation in a VOI within the cylindrical phantom for the investigated reconstruction methods. Note that GE data of the cylinder were reconstructed using both 4 iterations and 8 subsets (4i8s) and 10 iterations and 8 subsets (10i8s) for the DEW and TEW scatter correction methods, while Siemens data only were reconstructed using 10i8s for the DEW and TEW scatter correction methods. For the MC reconstruction, all data were reconstructed using 10i8s

System ID	Coefficient of variation		
	DEW	TEW	MC
GE 670	0.28 (4i8s) 0.53 (10i8s)	0.27 (4i8s) 0.60 (10i8s)	0.38
GE 870	0.23 (4i8s) 0.44 (10i8s)	0.28 (4i8s) 0.54 (10i8s)	0.28
Siemens Intevo 1	0.22	0.27	0.22
Siemens Intevo 2	0.20	0.26	0.20
Siemens Symbia 1	0.22	0.26	0.19
Siemens Symbia 2	0.21	0.26	0.20
Siemens Symbia 3	0.18	0.22	0.17

### Contrast recovery

**Fig. 5:**
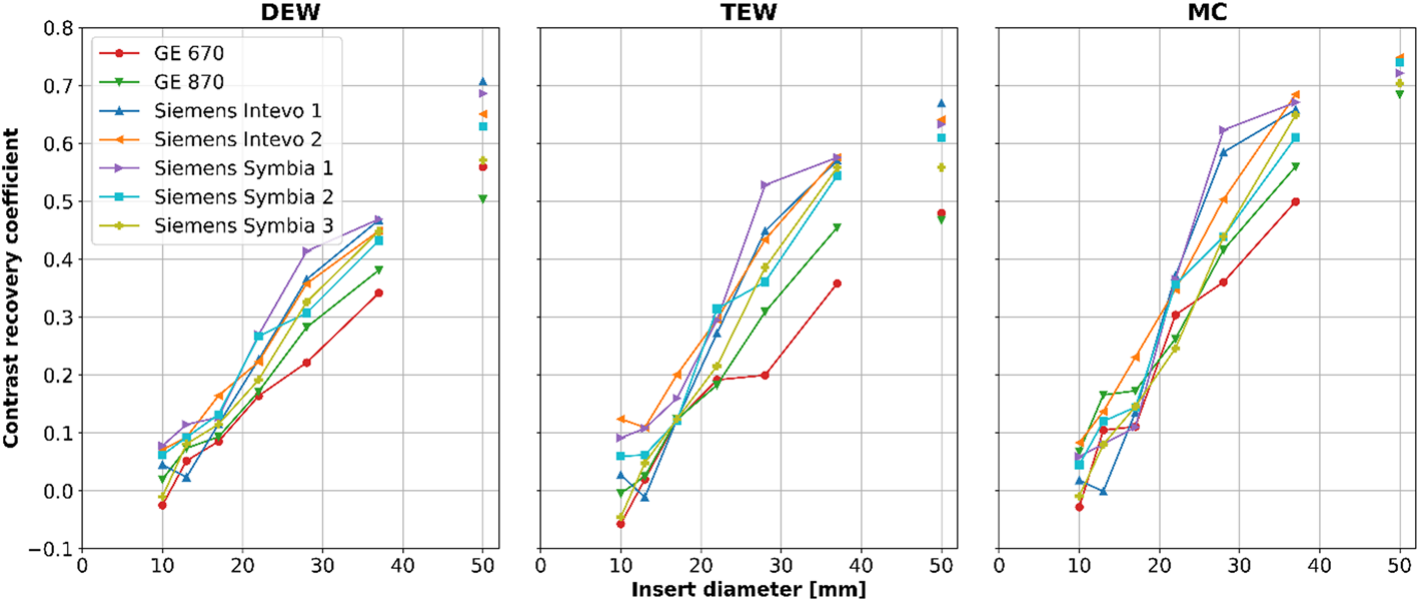
Mean CRC as a function of the diameter of the spherical (connected by a solid line) and lung inserts of the NEMA IEC phantoms measured on each scanner. All but GE 670 data have been averaged over the three consecutive acquisitions

The CRCs for all measurements of the NEMA IEC phantoms are presented in Fig. 5, and the mean ± SD (range) over all scanners, per scatter correction method, is reported in Table 5. When considering vendor-specific reconstructions, TEW reconstruction returned higher CRC values compared to DEW, with an average CRC increase of 23% for the largest sphere (mean taken over all measurements). MC reconstructions resulted in a further increase of the CRCs, but with a similar range as for the TEW reconstructions, highlighting that the scanner hardware (collimator) has a great impact on the achievable image quality, and in extension the achievable dosimetric accuracy. In addition, the mean ± SD (range) CRC of the measurements for which the standard NEMA IEC sphere configuration was used during the acquisition (thus excluding measurements from Siemens Intevo 1 and Siemens Symbia 1), per scatter correction method, is reported in the supplementary information.

**Table 5:** Mean ± SD (range) of all measurements of the CRCs for spheres and lung inserts of the NEMA IEC phantom, for the DEW, TEW, and MC reconstructions

NEMA IEC phantom insert	Contrast recovery coefficients mean ± SD (range)		
	DEW	TEW	MC
Sphere Ø 10 mm	0.04 ± 0.05 (−0.03–0.14)	0.04 ± 0.09 (−0.07–0.3)	0.04 ± 0.05 (−0.05–0.17)
Sphere Ø 13 mm	0.08 ± 0.04 (−0.01–0.15)	0.05 ± 0.05 (−0.05–0.17)	0.10 ± 0.06 (−0.02–0.18)
Sphere Ø 17 mm	0.12 ± 0.03 (0.06–0.22)	0.14 ± 0.04 (0.09–0.25)	0.15 ± 0.05 (0.08–0.28)
Sphere Ø 22 mm	0.22 ± 0.04 (0.15–0.29)	0.26 ± 0.06 (0.11–0.38)	0.32 ± 0.06 (0.23–0.41)
Sphere Ø 28 mm	0.34 ± 0.06 (0.22–0.45)	0.40 ± 0.10 (0.20–0.64)	0.49 ± 0.10 (0.31–0.67)
Sphere Ø 37 mm	0.44 ± 0.04 (0.34–0.51)	0.54 ± 0.07 (0.36–0.65)	0.62 ± 0.07 (0.45–0.72)
Lung insert Ø 50 mm (Ø 30 mm VOI)	0.62 ± 0.07 (0.45–0.71)	0.59 ± 0.07 (0.43–0.68)	0.73 ± 0.03 (0.65–0.77)

CRCs for the wider energy windows (main photopeak 20% width) resulted in lower CRCs for both the TEW and MC reconstructions than for the data presented in full here (main photopeak 15% width), see supplementary information.

### Contrast-to-noise ratio

**Fig. 6:**
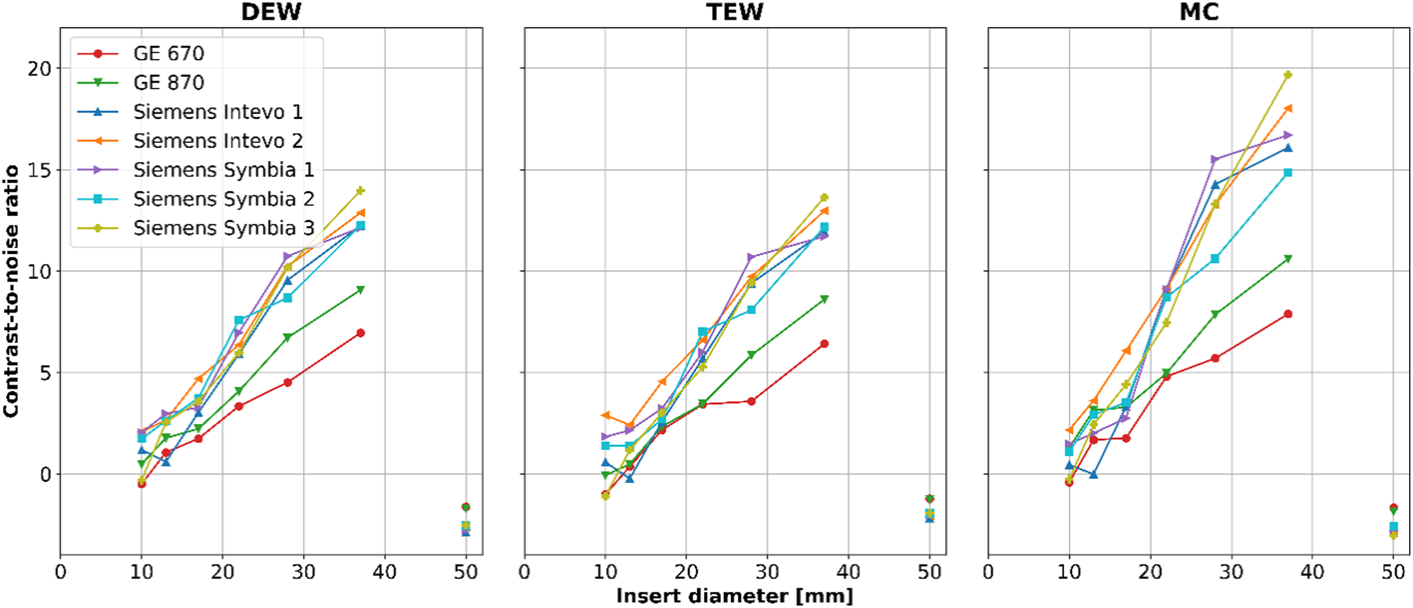
Mean CNR as a function of the diameter of the spherical (connected by a solid line) and lung inserts of the NEMA IEC phantoms measured at each scanner. All but GE 670 data have been averaged over the three consecutive acquisitions

CNRs for all measurements of the NEMA IEC phantoms are presented in Fig. 6, and the mean ± SD (range) over all scanners, per scatter correction method, is presented in Table 6. For the vendor-specific reconstructions (DEW and TEW) the CNRs are similar when comparing each scanner separately, meaning that hot and/or cold objects are equally easy to detect in images using either of these scatter correction methods. The vendor-neutral MC reconstruction algorithm results in increased CNRs compared to the vendor-specific algorithms, however the range of the CNRs also generally increases. This indicates that scanner (or vendor) specific reconstruction settings are needed to be able to harmonize the image quality for vendor-neutral reconstruction software. In addition, the mean ± SD (range) CNR of the measurements for which the standard NEMA IEC sphere configuration was used during the acquisition (thus excluding measurements from Siemens Intevo 1 and Siemens Symbia 1), per scatter correction method, is reported in the supplementary information.

**Table 6:** Mean ± SD (range) of all measurements of the CNRs for spheres and lung inserts of the NEMA IEC phantom, for the DEW, TEW, and MC reconstructions

NEMA IEC phantom insert	Contrast−to−noise ratio mean ± SD (range)		
	DEW	TEW	MC
Sphere Ø 10 mm	1.11 ± 1.30 (−1.03–3.94)	0.82 ± 1.97 (−1.77–7.13)	0.89 ± 1.27 (−1.57–4.47)
Sphere Ø 13 mm	2.13 ± 1.14 (−0.24–4.15)	1.18 ± 1.04 (−1.08–3.45)	2.28 ± 1.27 (−0.52–4.44)
Sphere Ø 17 mm	3.33 ± 1.06 (1.36–6.36)	3.02 ± 1.01 (1.62–5.95)	3.67 ± 1.35 (1.24–7.13)
Sphere Ø 22 mm	5.99 ± 1.32 (3.34–8.23)	5.55 ± 1.48 (2.19–8.48)	7.75 ± 1.93 (3.9–10.68)
Sphere Ø 28 mm	9.09 ± 1.87 (4.51–12.16)	8.58 ± 2.24 (3.57–13.28)	11.79 ± 3.31 (4.67–16.59)
Sphere Ø 37 mm	11.82 ± 1.96 (6.95–14.46)	11.55 ± 2.16 (6.41–14.43)	15.18 ± 3.71 (6.67–20.25)
Lung insert Ø 50 mm (Ø 30 mm VOI)	−2.47 ± 0.43(−3.01–−1.53)	−1.87 ± 0.33(−2.22–−1.15)	−2.56 ± 0.48(−3.08–−1.51)

No systematic increase in the CNRs for the TEW and MC reconstructions was observed when considering the wider energy window (main photopeak 20% width) compared to the 15% window width presented here, see supplementary information.

## Discussion

In the presented study, scanner sensitivity and image quality of ^166^Ho-SPECT/CT scans were quantitively evaluated for two different vendors and a total of seven scanners. Based on the vendor-specific reconstructions it was shown that TEW-based scatter correction improves the quantitative accuracy (higher CRCs) of the images (Fig. 5), compared to DEW-based scatter correction, while the detectability of high intensity objects is unaffected (Fig. 6).

### Harmonization

Given a fixed activity level and imaging time, it is challenging to fully harmonize image quality of scans acquired with SPECT/CT scanners from different vendors. The presented scanner sensitivities already indicate that scanner hardware has a large influence on the count statistics, which directly affects the noise level in the reconstructed images. The most prominent difference in the hardware between the two vendors included in this study were the longer holes of the GE MEGP collimator (58 mm) compared to the Siemens MELP collimator (40.64 mm), see Table 1. This collimator characteristic led to a higher fraction of (photopeak) photons being rejected for MEGP than for MELP, and therefore induced a lower measured sensitivity (Table 3). The longer collimator holes should however also lead to a higher resolution for the MEGP than MELP. Given a longer imaging time (increased count statistics), this characteristic would results in increased CRCs for the MEGP compared to the MELP.

Further, when choosing settings for the image reconstruction there is always a trade-off between increasing the contrast, i.e. iterating longer, and restraining the image noise, i.e. limiting the number of updates. Image quality optimization is ultimately about finding the balance between the increase in the CRC and the increase in the noise (COV). To carry out accurate SPECT-based dosimetry in a multi-scanner setting, we consider CRC to be the most important parameter to be harmonized, as it predicts the accuracy of the activity recovery in tumors. Harmonizing the images for dosimetry therefore means that the range of the CRCs is minimized in order to get reproducible results across different scanners. The vendor-neutral reconstructions demonstrated that equal reconstruction settings do not automatically lead to comparable image quality. To further harmonize the images, scanner-specific post-reconstruction filters could potentially be applied to reduce the image noise and thereby increase the CNRs. However, one should be aware that a gain in CNRs through filtering comes at a cost of decreased CRCs, see the supplementary information.

Within this project SPECT data were also acquired for a wider photopeak (centered at 81 keV, 20% width), and adjacent lower and upper scatter windows (10% width), on six of the seven scanners. These data have been reconstructed utilizing the TEW and MC scatter correction methods (10 iterations and 8 subsets independent of the scanner used for the acquisition). A decrease of the CRCs from the wider photopeak was observed (supplementary information) compared to the images reconstructed based the narrower photopeak data (Fig. 5). Nevertheless, an advantage of the wider photopeak window is that, since more counts are measured, the noise in the images is intrinsically lower than for the narrower photopeak window, resulting in comparable CNRs. Additionally, the wider photopeak window could also be more robust to peaking or energy calibration inaccuracies, since a larger portion of the primary photopeak photons are registered within the main photopeak window.

### Scatter correction

The suitability of the scatter correction methods was evaluated based on the uniformity of a line profile through the cylindrical phantom. Comparing the two methods utilized in the vendor-specific software, we conclude that TEW-based scatter correction is superior to the DEW-based scatter correction, in accordance with [17], as shown by the flatter line profile (Fig. 4).

Furthermore, the TEW-based images resulted in higher CRCs compared to the DEW-based images (Fig. 5). However, since images reconstructed with the DEW method contained more photopeak scatter (Fig. 4), the lower CRCs measured for the DEW is likely to be dominated by higher background signal rather than a lower reconstructed sphere activity.

Irrespective of the scanner, the CNRs based on the DEW- and TEW-based scatter corrections were comparable: the gain in contrast for the TEW method over the DEW method was counteracted by a similar increase in noise level.

### Convergence

For data acquired with Siemens Intevo 1 and Siemens Symbia 1 (blue and purple lines in Fig. 5, respectively), the CRCs for the second largest sphere (Ø 28 mm) were systematically higher (across DEW, TEW and MC) than those of the other phantom/scanner combinations. This behavior was likely due to the spheres being positioned differently, i.e. with the Ø 28 mm sphere closer to the phantom wall, and therefore closer to the detectors compared to the standard NEMA configuration used for performing the measurements on all the other scanners [23], see Fig. 2. This characteristic of SPECT/CT imaging has previously been demonstrated for lutetium-177 [24] and it emphasizes that not only the size of an imaged object, but also its spatial position within a scatter medium/patient's body influences the achievable convergence and the measured activity recovery.

Additionally, the sphere sizes present in the standard NEMA IEC phantom are not large enough for curves in Fig. 5 (CRCs versus sphere diameter) to sufficiency reach a plateau. This means that the quantification accuracy of the spheres (CRCs) are heavily affected by partial volume effects. Since the CRC-curves are very steep for the range of spheres present in the NEMA phantom, this indicates that the measured values are sensitive to resolution differences between the SPECT scanners and that can hamper the harmonization of this parameter. The implication for clinical dosimetry is that the application of partial volume correction methods, such as (harmonized) recovery curves [22, 25], is a complex endeavor with many sources of uncertainties. As an alternative, correction methods relying on the dilation of the VOI, to encompass the spill-out, could be considered [21].

### Limitations

Among the GE scanners, higher noise, and lower CRCs and CNRs was measured with the GE 670-scanner, compared to the GE 870-scanner, despite the comparable hardware (Table 1). One difference between these two scanners was, however, that a dedicated uniformity map had been made for ^166^Ho before acquiring the data for GE 870. Also, only one dataset of the NEMA IEC phantom was collected for GE 670, which could have been an outlier.

Additionally, this study was focused on conventional SPECT/CT scanners with two NaI detectors, and did not include any scanners featuring a triple-headed setup, cadmium-zinc-telluride detectors or scanners with ring-shaped geometry. The image quality of vendor-specific quantitative reconstruction software was also not investigated.

Furthermore, to limit the scope of this study, a single activity level of approximately 300 MBq was chosen for the SPECT imaging. This activity level was chosen to match the QuiremScout™ [20] as the pre-treatment dosimetry in ^166^Ho radioembolization is based on this. The impact of a large activity range (approximately 60–3100 MBq) on the SPECT scanner linearity, detector response, and image quality has previously been investigated in [21, 22].

### Towards clinical ^166^Ho-SPECT-based dosimetry

In this study, we paved the way for ^166^Ho-SPECT image quality harmonization, presenting multi-center, multi-vendor data acquired with protocols readily available to be implemented in clinical practice. However, to be able to calculate the absorbed dose and determine dose–response relationships, the images need to be quantitative. Currently, some guidelines recommend the use of a scanner-specific conversion/calibration factor for this quantification step [3, 26], and vendors are introducing absolute quantification methods to the market [27, 28]. Note that the accuracy of both of these quantification methods depend on the activity present in the scanner at the time of the acquisition, in particular non-linear detector response and high count rate (high dead-time) reduces the dosimetric accuracy significantly [22].

One major advantage for dosimetry of ^166^Ho radioembolization, compared to dosimetry of systemic therapies or even technetium-99m macroaggregated albumin (^99m^Tc-MAA) scout, is that most of the injected particles lodge in the liver and are retained there permanently (^166^Ho microspheres are considered to have no biological half-life). With a single time-point, single bed-position SPECT of the liver and part of the lungs, the image can be quantified based on the known injected activity and converted to absorbed dose [21, 22, 29, 30]. To reach a consensus on how to perform ^166^Ho-SPECT/CT-based dosimetry, the accuracy of the available quantification methods needs to be investigated for ^166^Ho.

## Conclusions

The use of TEW-based scatter correction for ^166^Ho-SPECT reconstructions is recommended over the (to date) most clinically used DEW method, as it improves image contrast and uniformity. Based on the system sensitivity measurements, and the vendor-neutral reconstruction software, this study demonstrated that identical parameters do not yield harmonized image quality for data originating from scanners of different vendors, likely due to differences in collimator characteristics. Lastly, the data presented in this study could serve as a benchmark when setting up ^166^Ho-SPECT acquisition and reconstruction protocols in other hospitals.

## Supplementary Information

Additional file1 (DOCX 2333 KB)

## Data Availability

The scripts for the analyses are available online at https://bitbucket.org/MedPhysNL/hoha-analysis. The datasets can be made available from the corresponding author on reasonable request.

## Abbreviations

^99m^Tc-MAA: Technetium-99m macroaggregated albumin
^166^Ho: Holmium-166
COV: Coefficient of variation
CNR: Contrast-to-noise ratio
CRC: Contrast recovery coefficient
CT: Computed tomography
DEW: Dual-energy window scatter correction
EANM: European association of nuclear medicine
EARL: EANM Research4Life GmbH
LUMC: Leiden University Medical Center
MC: Monte Carlo
MEGP: Medium energy general purpose
MELP: Medium energy low penetration
MUMC +: Maastricht University Medical Center
NKI: The Netherlands Cancer Institute
PET: Positron emission tomography
Radboudumc: Radboud University Medical Center
SD: Standard deviation
SPECT: Single photon emission computed tomography
TEW: Triple-energy window scatter correction
UMCU: University Medical Center Utrecht
VOI: Volume of interest
