# Supplementary material for: Towards harmonized holmium-166 SPECT image quality for dosimetry: a multi-center, multi-vendor study
Source: EJNMMI Phys. 2025 Mar 19;12:24. doi: 10.1186/s40658-025-00733-8 (PMC11920561; doi:10.1186/s40658-025-00733-8)
Supplement: Supplementary file 1 — Additional file1 (DOCX 2333 KB) [file 40658_2025_733_MOESM1_ESM.docx]

Supplementary information for:

# Towards harmonized holmium-166 SPECT image quality for dosimetry: a multi-center, multi-vendor study

EJNMMI Physics

### Authors:

Lovisa E.L. Westlund Gotby*†^1^, Martina Stella*^2^, Camille D.E. Van Speybroeck^1^, Daphne Lobeek^1^, Floris H.P. van Velden^3^, Mette K. Stam^3^, Petra Dibbets-Schneider^3^, Daphne M.V. de Vries-Huizing^4^, Erik-Jan Rijkhorst^4^, Berlinda J. de Wit-van de Veen^4^, Roel Wierts^5^, Rob van Rooij^2^

^*^These authors contributed equally to this work and share first authorship

†Corresponding author at [Lovisa.WestlundGotby@radboudumc.nl](mailto:Lovisa.WestlundGotby@radboudumc.nl)

### Affiliations:

^1^Department of Medical Imaging, Radboud University Medical Center, Geert Grooteplein Zuid 10, 6525 GA, Nijmegen, The Netherlands

^2^Department of Radiology and Nuclear Medicine, University Medical Centre Utrecht, Heidelberglaan 100, 3584 CX Utrecht, The Netherlands

^3^Department of Radiology, Leiden University Medical Center, Section of Nuclear Medicine, Albinusdreef 2, 2333 ZA, Leiden, The Netherlands

^4^Department of Nuclear Medicine, Netherlands Cancer Institute, Plesmanlaan 121, Amsterdam 1066 CX, The Netherlands

^5^Department of Radiology and Nuclear medicine, Maastricht University Medical Center, P. Debyelaan 25, 6299 HX Maastricht, The Netherlands

# Supplementary information

## Main photopeak 15% width

### Contrast recovery

The mean ± SD (range) of the CRCs for the measurements in which the standard NEMA IEC sphere configuration has been used is presented in Table S.1. This includes the data from GE 670, GE 870, Siemens Intevo 2, Siemens Symbia 2, and Siemens Symbia 3, see Figure 2 in the main text for the sphere configuration.

The largest difference in the mean CRC values reported here, compared to the data in the main text (Table 5), is a decrease of the mean CRC for the three largest spheres. The reason for this discrepancy is both that the positioning of these spheres in the excluded data sets (Siemens Intevo 1 and Siemens Symbia 1) were advantageous in terms of measuring high CRCs (positive bias of the mean CRCs in the main text), as well as the mean now being more influenced by the GE scanners (negative bias of the mean CRCs; 4 out of 13 measurements (~31%) originating from GE scanners compared to 4 out of 19 measurements (~21%) in the main text).

Table S.1 The mean ± SD (range) CRC for spheres and lung inserts of the measurements for which the standard NEMA IEC sphere configuration was used during the acquisition (thus excluding measurements from Siemens Intevo 1 and Siemens Symbia 1). The DEW, TEW, and MC reconstructions are presented in the different columns

| **NEMA IEC phantom insert** | **Contrast recovery coefficients**  **mean ± SD (range)** | | |
| --- | --- | --- | --- |
|  | DEW | TEW | MC |
| **Sphere Ø 10 mm** | 0.03 ± 0.05  (-0.03 – 0.14) | 0.03 ± 0.10  (-0.07 – 0.30) | 0.04 ± 0.06  (-0.05 – 0.17) |
| **Sphere Ø 13 mm** | 0.08 ± 0.03  (0.04 – 0.13) | 0.06 ± 0.04  (0.02 – 0.17) | 0.12 ± 0.04  (0.06 – 0.18) |
| **Sphere Ø 17 mm** | 0.12 ± 0.04  (0.06 – 0.22) | 0.14 ± 0.05  (0.09 – 0.25) | 0.16 ± 0.05  (0.08 – 0.28) |
| **Sphere Ø 22 mm** | 0.21 ± 0.04  (0.15 – 0.29) | 0.25 ± 0.07  (0.11 – 0.38) | 0.30 ± 0.05  (0.23 – 0.40) |
| **Sphere Ø 28 mm** | 0.31 ± 0.05  (0.22 – 0.38) | 0.36 ± 0.08  (0.20 – 0.46) | 0.44 ± 0.07  (0.31 – 0.52) |
| **Sphere Ø 37 mm** | 0.42 ± 0.04  (0.34 – 0.46) | 0.52 ± 0.07  (0.36 – 0.61) | 0.61 ± 0.07  (0.45 – 0.70) |
| **Lung insert Ø 50 mm (Ø 30 mm VOI)** | 0.59 ± 0.06  (0.45 – 0.67) | 0.56 ± 0.07  (0.43 – 0.66) | 0.72 ± 0.03  (0.65 – 0.77) |

### Contrast-to-noise ratio

The mean ± SD (range) of the CNRs for the measurements for which the standard NEMA IEC sphere configuration has been used is presented in Table S.2. This includes the data from GE 670, GE 870, Siemens Intevo 2, Siemens Symbia 2, and Siemens Symbia 3, see Figure 2 in the main text for the sphere configuration.

Similar to the results for the CRCs, a decrease in the CNRs for the three largest spheres is observed for the data reported here compared to the data reported in the main text.

Table S.2 The mean ± SD (range) CNR for spheres and lung inserts of the measurements for which the standard NEMA IEC sphere configuration was used during the acquisition (thus excluding measurements from Siemens Intevo 1 and Siemens Symbia 1). The DEW, TEW, and MC reconstructions are presented in the different columns

| **NEMA IEC phantom insert** | **Contrast-to-noise ratio**  **mean ± SD (range)** | | |
| --- | --- | --- | --- |
|  | DEW | TEW | MC |
| **Sphere Ø 10 mm** | 0.87 ± 1.41  (-1.03 – 3.94) | 0.64 ± 2.28  (-1.77 – 7.13) | 0.86 ± 1.41  (-1.57 – 4.47) |
| **Sphere Ø 13 mm** | 2.29 ± 0.97  (0.92 – 4.15) | 1.28 ± 0.84  (0.36 – 3.45) | 2.83 ± 0.88  (0.96 – 4.44) |
| **Sphere Ø 17 mm** | 3.41 ± 1.25  (1.36 – 6.36) | 3.08 ± 1.17  (1.62 – 5.95) | 3.95 ± 1.48  (1.24 – 7.13) |
| **Sphere Ø 22 mm** | 5.79 ± 1.49  (3.34 – 8.23) | 5.42 ± 1.63  (2.19 – 8.48) | 7.17 ± 1.97  (3.90 – 10.68) |
| **Sphere Ø 28 mm** | 8.60 ± 1.92  (4.51 – 11.22) | 7.90 ± 2.22  (3.57 – 10.53) | 10.46 ± 3.01  (4.67 – 14.55) |
| **Sphere Ø 37 mm** | 11.65 ± 2.28  (6.95 – 14.46) | 11.43 ± 2.45  (6.41 – 14.43) | 14.66 ± 4.30  (6.67 – 20.25) |
| **Lung insert Ø 50 mm (Ø 30 mm VOI)** | -2.31 ± 0.43  (-2.71 – -1.53) | -1.76 ± 0.35  (-2.15 – -1.15) | -2.44 ± 0.53  (-3.08 – -1.51) |

## Main photopeak 20% width

In addition to the acquisition and reconstruction protocols with a main photopeak window width of 15% (main text), data with a main photopeak window width of 20%, also centered at 81 keV, were acquired to assess the impact of the window width on the triple energy window (TEW) and Monte Carlo (MC) scatter correction methods. The lower and upper scatter windows were positioned directly adjacent to the main photopeak and had a width of 10%. The same metrics presented in the main text were used for evaluation of this data. Contrary to reconstructions that were presented in the main text, all the vendor-specific reconstructions were performed using 10 iterations and 8 subsets. Because of logistical issues, no data for the 20% photopeak window were acquired for GE 670.

The energy spectrum for the NEMA IEC phantom acquired with both MELP and MEGP collimators is presented in Figure S.1. The wider photopeak window ensures that the counts in the main photopeak are acquired as main photopeak counts (see purple window in Figure S.1). However, the counts generated by scattered photons in a medium (e.g. NEMA IEC phantom) can also be included in the main photopeak window. Hence, there is a trade-off between increasing the main photopeak window width, and thereby increasing the system sensitivity, and increasing the risk of the main photopeak window being contaminated by scattered photons.

In general, the 20% main photopeak window width (and adjacent lower and upper scatter windows with 10% width) resulted in lower noise and higher system sensitivity than the data presented in the main text (15% main photopeak width). However, lower CRCs and roughly unchanged CNRs were also measured. The protocol with wider energy window width is feasible and has the advantage of being less susceptible to peak shift. For the purpose of this study, the 15% photopeak width acquisition protocol was presented in the main as it compares more readily with the (to date) most clinically used DEW protocol (also featuring a 15% main photopeak window). Furthermore, with the aim of quantitative SPECT images for ^166^Ho dosimetry, the higher CRCs of the 15% photopeak width protocol were considered most relevant.


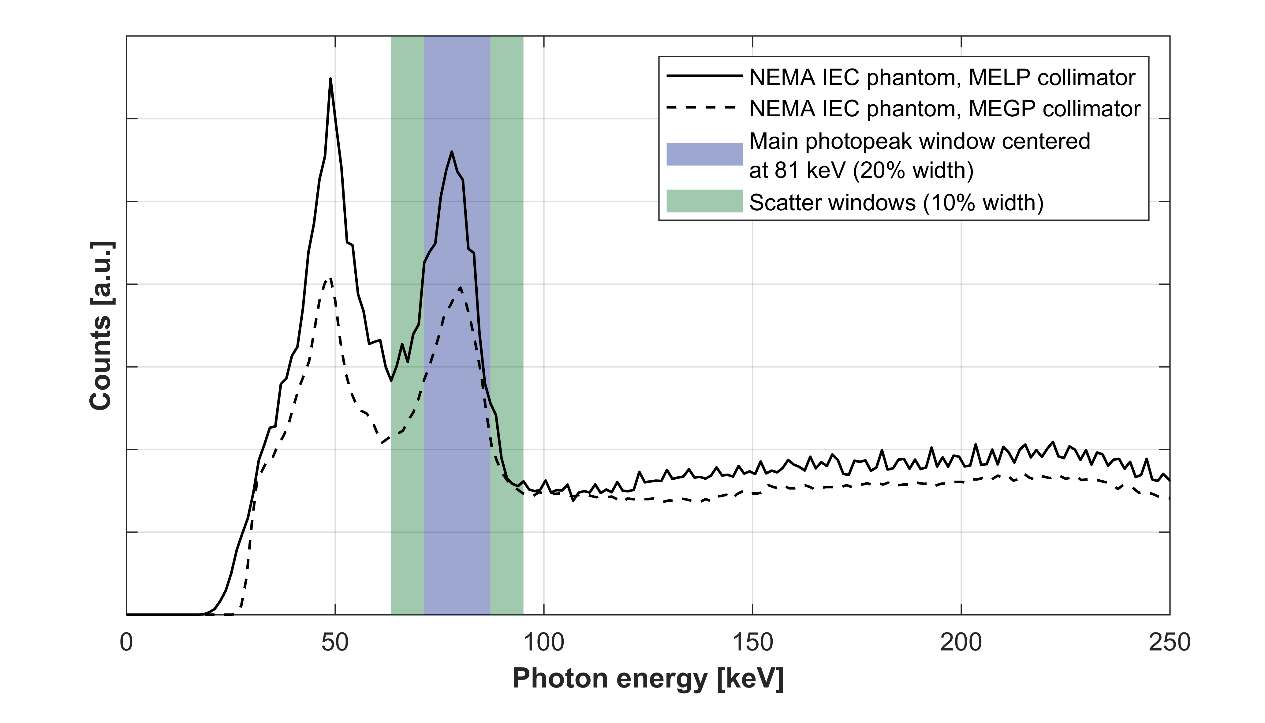


Figure S.1 Energy spectra and acquired photon energy windows. ^166^Ho-spectra for the NEMA IEC phantom acquired with the Siemens MELP (solid black line) and the GE MEGP (dashed black line) collimators are shown. The NEMA spectra were normalized and scaled the same way as in the main text. The main photopeak window is displayed in purple, and the adjacent scatter windows are displayed in green (used for the TEW reconstructions)

### System sensitivity

System sensitivity based on data from cylindrical phantom, measured as cps/MBq, in each acquired energy window is reported in Table S.3. The sensitivity for the main photopeak with 20% window width on average increased by 23%, compared to photopeak with 15% width. Similar to the results reported in the main text, data acquired with the GE MEGP collimators resulted in approximately 33% lower system sensitivity in the main photopeak window compared to data acquired with the Siemens MELP collimators.

Table S.3 System sensitivity [cps/MBq] per acquired energy window for each of the SPECT/CT systems included in this study. To facilitate comparison with the system sensitivities from the main text, these numbers are shown in italic font style

| **System ID** | **Low scatter window [cps/MBq]** | | **Main photopeak window at 81 keV [cps/MBq]** | | **High scatter window [cps/MBq]** | |
| --- | --- | --- | --- | --- | --- | --- |
|  | **10% width** | ***8% width*** | **20% width** | ***15% width*** | **10% width** | ***8% width*** |
| GE 670 | - | *2.16* | - | *4.54* | - | *1.42* |
| GE 870 | 2.10 | *2.00* | 5.51 | *4.50* | 1.42 | *1.29* |
| Siemens Intevo 1 | 2.94 | *2.70* | 8.18 | *6.64* | 1.57 | *1.57* |
| Siemens Intevo 2 | 3.19 | *2.96* | 8.70 | *7.04* | 1.81 | *1.79* |
| Siemens Symbia 1 | 2.91 | *2.68* | 8.00 | *6.53* | 1.59 | *1.63* |
| Siemens Symbia 2 | 3.11 | *2.85* | 8.72 | *7.03* | 1.87 | *1.89* |
| Siemens Symbia 3 | 3.43 | *3.15* | 9.57 | *7.72* | 2.05 | *2.09* |

### Uniformity in cylindrical phantom

Line profiles, representing image uniformity, are displayed in Figure S.2 for each scanner and for each of the two scatter correction methods presented (TEW and MC). Averaged over all scanners, the TEW reconstructions resulted in an $A$ coefficient of 0.00 ± 0.17 (mean ± SD), and MC in 0.10 ± 0.17.


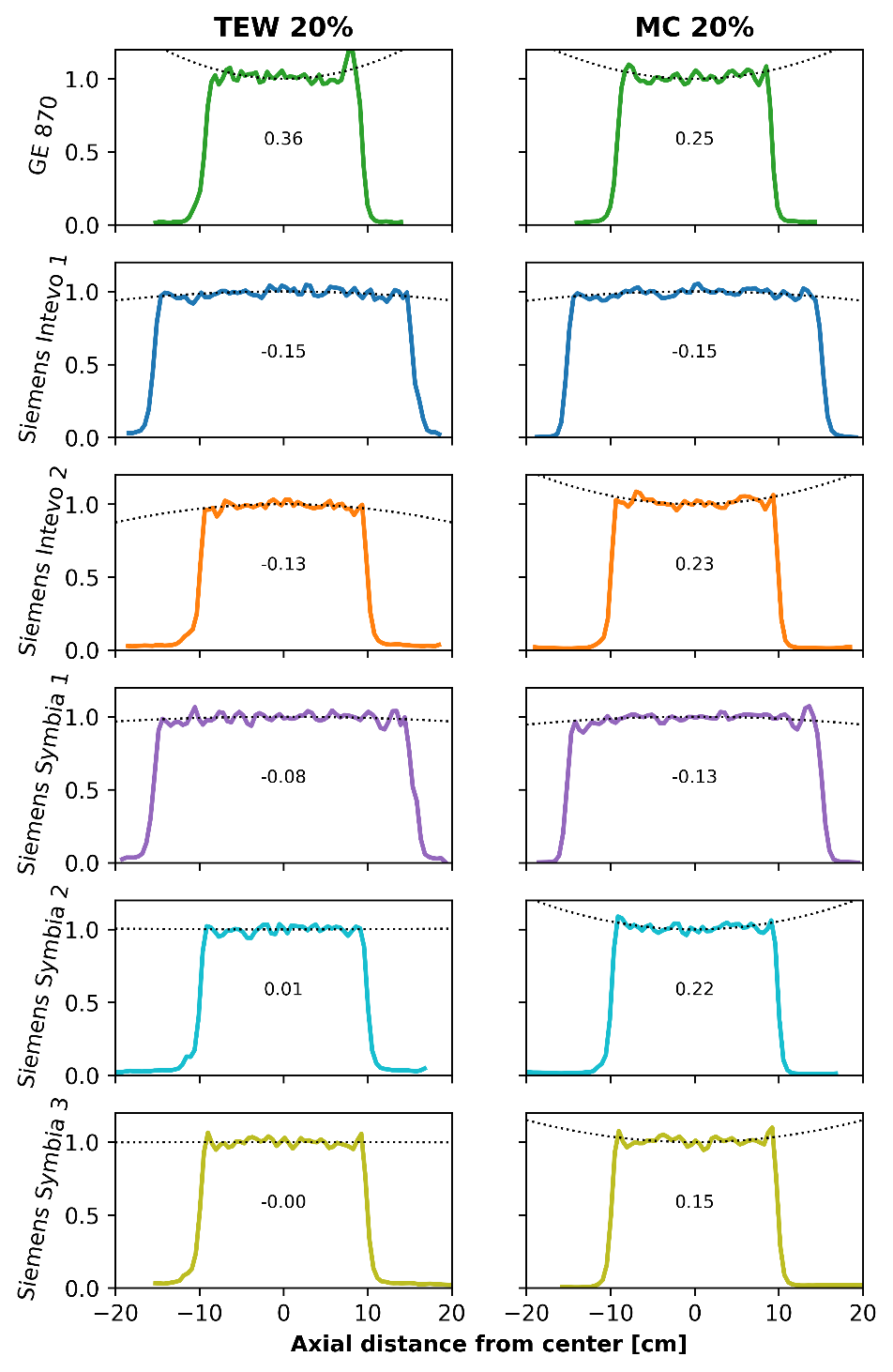


Figure S.2 Line profiles measured along the axial direction (along the length) of the cylindrical phantom acquired for each scanner. The dotted lines indicate the fitted curvature along the tops of the line profiles (extended outwards for sake of clarity). For each plot, the curvature coefficient A is reported

### Noise

Noise, measured as the mean of coefficient of variation per slice in a VOI within the cylindrical phantom (same VOI as in the main text) for the data acquired with the wider energy windows is presented in Table S.4. Note that all data were reconstructed with 10 iterations and 8 subsets. On average the COV decreases with 7% for TEW reconstructs when comparing the 20% photopeak window width (presented here) with the 15% photopeak window width (main text). For MC reconstructions, the average COV decrease was 5%.

Table S.4 Noise, measured as the coefficient of variation in a VOI within the cylindrical phantom for the investigated reconstruction methods

| **System ID** | **Coefficient of variation** | |
| --- | --- | --- |
|  | **TEW 20%** | **MC 20%** |
| GE 670 | - | - |
| GE 870 | 0.50 | 0.28 |
| Siemens Intevo 1 | 0.25 | 0.20 |
| Siemens Intevo 2 | 0.25 | 0.19 |
| Siemens Symbia 1 | 0.25 | 0.19 |
| Siemens Symbia 2 | 0.24 | 0.19 |
| Siemens Symbia 3 | 0.19 | 0.15 |

### Contrast recovery

CRCs are depicted in Figure S.3 and values are reported in Table S.5. In general, CRCs for the wider energy windows (main photopeak 20% width) resulted in lower CRCs for both the TEW and MC reconstructions than for the data acquired with main photopeak with 15% width. For the reconstructions of the Siemens data, the CRC of the largest sphere on average decreased with 12% for TEW and 8% for MC, when comparing the wider window with the narrower window.

In addition, the mean ± SD (range) CRC of the measurements for which the standard NEMA IEC sphere configuration was used during the acquisition (thus excluding measurements from Siemens Intevo 1 and Siemens Symbia 1), per scatter correction method, is reported in Table S.6. Similarly to the results reported for the main photopeak with a 15% width, a slight decrease in the mean CRC for the three largest spheres can be observed when comparing Table S.6 with Table S.5. On the contrary, a slight increase is observed for the three smallest spheres for the same comparison.


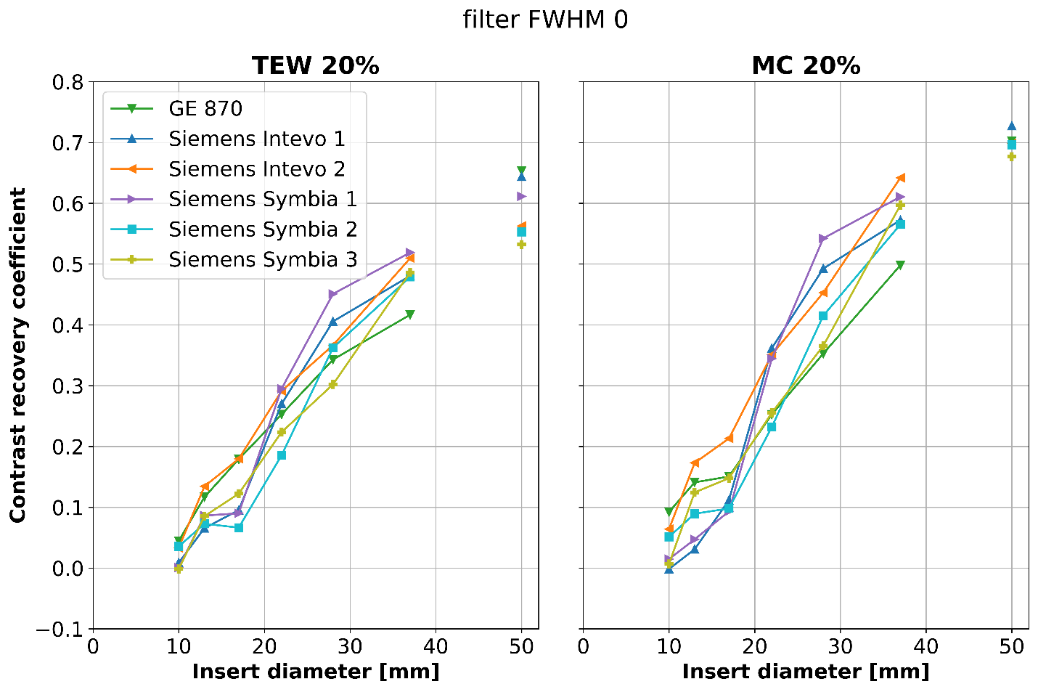


Figure S.3 CRCs as a function of the diameter of the spherical and lung inserts of the NEMA IEC phantom measured on each scanner. All data have been averaged over the three consecutive acquisitions

Table S.5 Mean ± SD (range) of the CRCs for spheres and lung inserts of the NEMA IEC phantom, for the TEW, and the MC reconstructions

| **NEMA IEC phantom insert** | **Contrast recovery coefficients**  **mean ± SD (range)** | |
| --- | --- | --- |
|  | TEW 20% | MC 20% |
| **Sphere Ø 10 mm** | 0.02 ± 0.04  (-0.03 – 0.12) | 0.04 ± 0.05  (-0.02 – 0.16) |
| **Sphere Ø 13 mm** | 0.09 ± 0.04  (0.03 – 0.17) | 0.10 ± 0.06  (-0.0 – 0.18) |
| **Sphere Ø 17 mm** | 0.12 ± 0.05  (0.02 – 0.21) | 0.14 ± 0.05  (0.05 – 0.22) |
| **Sphere Ø 22 mm** | 0.25 ± 0.05  (0.17 – 0.35) | 0.30 ± 0.07  (0.22 – 0.43) |
| **Sphere Ø 28 mm** | 0.37 ± 0.05  (0.27 – 0.49) | 0.44 ± 0.07  (0.32 – 0.6) |
| **Sphere Ø 37 mm** | 0.48 ± 0.04  (0.38 – 0.53) | 0.58 ± 0.05  (0.46 – 0.66) |
| **Lung insert Ø 50 mm (Ø 30 mm VOI)** | 0.56 ± 0.05  (0.49 – 0.68) | 0.70 ± 0.02  (0.67 – 0.74) |

Table S.6 The mean ± SD (range) CRC for spheres and lung inserts of the measurements for which the standard NEMA IEC sphere configuration was used during the acquisition (thus excluding measurements from Siemens Intevo 1 and Siemens Symbia 1). The DEW, TEW, and MC reconstructions are presented in the different columns

| **NEMA IEC phantom insert** | **Contrast recovery coefficients**  **mean ± SD (range)** | |
| --- | --- | --- |
|  | TEW 20% | MC 20% |
| **Sphere Ø 10 mm** | 0.03 ± 0.05  (-0.03 – 0.12) | 0.05 ± 0.05  (0.00 – 0.16) |
| **Sphere Ø 13 mm** | 0.10 ± 0.04  (0.03 – 0.17) | 0.13 ± 0.04  (0.05 – 0.18) |
| **Sphere Ø 17 mm** | 0.14 ± 0.06  (0.02 – 0.21) | 0.15 ± 0.05  (0.08 – 0.22) |
| **Sphere Ø 22 mm** | 0.24 ± 0.05  (0.17 – 0.34) | 0.27 ± 0.05  (0.22 – 0.37) |
| **Sphere Ø 28 mm** | 0.34 ± 0.03  (0.27 – 0.39) | 0.40 ± 0.05  (0.32 – 0.47) |
| **Sphere Ø 37 mm** | 0.47 ± 0.04  (0.38 – 0.52) | 0.58 ± 0.06  (0.46 – 0.66) |
| **Lung insert Ø 50 mm (Ø 30 mm VOI)** | 0.58 ± 0.05  (0.49 – 0.68) | 0.69 ± 0.02  (0.67 – 0.73) |

### Contrast-to-noise ratio

CNRs are depicted in Figure S.4 and values are reposted in Table S.7. No systematic change in the CNRs (all spheres) for the TEW and MC reconstructions was observed when considering the wider energy window (main photopeak 20% width) compared to the 15% window width presented in the main text. For reconstructions based on the Siemens data, the average CNR of the largest sphere decreased with 5% and 3% for TEW and MC reconstructions, respectively, when comparing the wider energy window with the data in the main text.

In addition, the mean ± SD (range) CNR of the measurements for which the standard NEMA IEC sphere configuration was used during the acquisition (thus excluding measurements from Siemens Intevo 1 and Siemens Symbia 1), per scatter correction method, is reported in Table S.8. Similarly to the results for the CRCs of the main photopeak window with a 20% width, a slight decrease is observed for the three largest spheres when comparing Table S.8 with Table S.7, while a slight increase is observed for the three smallest spheres.


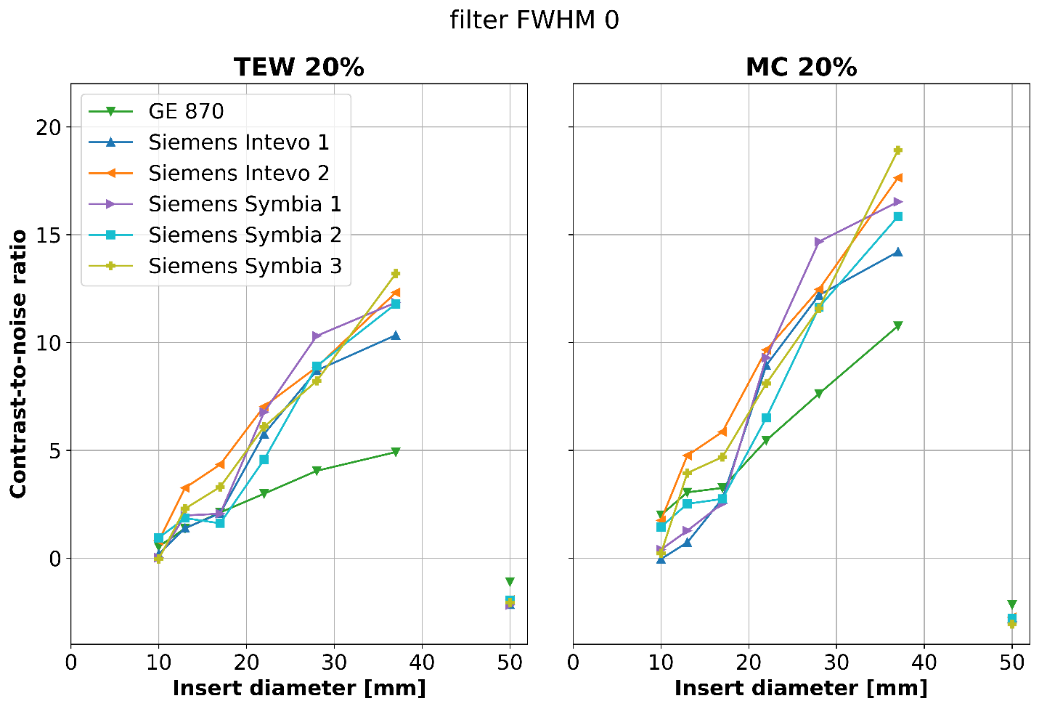


Figure S.4 CNRs as a function of the diameter of the spherical and lung inserts of the NEMA IEC phantoms measured on each scanner. All data have been averaged over the three consecutive acquisitions

Table S.7 Mean ± SD (range) of the CNRs for spheres and lung inserts of the NEMA IEC phantom, for the TEW, and the MC reconstructions

| **NEMA IEC phantom insert** | **Contrast-to-noise ratio**  **mean ± SD (range)** | |
| --- | --- | --- |
|  | TEW 20% | MC 20% |
| Sphere Ø 10 mm | 0.40 ± 0.9  (-0.67 – 2.97) | 0.96 ± 1.12  (-0.56 – 3.5) |
| Sphere Ø 13 mm | 2.02 ± 0.92  (0.59 – 4.18) | 2.71 ± 1.54  (-0.02 – 4.99) |
| Sphere Ø 17 mm | 2.58 ± 1.23  (0.49 – 5.04) | 3.64 ± 1.38  (1.33 – 6.04) |
| Sphere Ø 22 mm | 5.53 ± 1.64  (2.28 – 8.24) | 7.99 ± 1.91  (4.78 – 11.44) |
| Sphere Ø 28 mm | 8.17 ± 2.06  (3.53 – 11.55) | 11.69 ± 2.28  (6.98 – 16.29) |
| Sphere Ø 37 mm | 10.73 ± 2.82  (4.49 – 14.19) | 15.65 ± 2.78  (10.08 – 19.22) |
| Lung insert Ø 50 mm (Ø 30 mm VOI) | -1.90 ± 0.38  (-2.27 – -1.02) | -2.75 ± 0.30  (-3.14 – -2.08) |

Table S.8 The mean ± SD (range) CNR for spheres and lung inserts of the measurements for which the standard NEMA IEC sphere configuration was used during the acquisition (thus excluding measurements from Siemens Intevo 1 and Siemens Symbia 1). The DEW, TEW, and MC reconstructions are presented in the different columns

| **NEMA IEC phantom insert** | **Contrast-to-noise ratio**  **mean ± SD (range)** | |
| --- | --- | --- |
|  | TEW 20% | MC 20% |
| Sphere Ø 10 mm | 0.56 ± 1.04  (-0.67 – 2.97) | 1.35 ± 1.14  (0.07 – 3.50) |
| Sphere Ø 13 mm | 2.19 ± 1.04  (0.59 – 4.18) | 3.56 ± 1.03  (1.46 – 4.99) |
| Sphere Ø 17 mm | 2.84 ± 1.35  (0.49 – 5.04) | 4.13 ± 1.36  (2.21 – 6.04) |
| Sphere Ø 22 mm | 5.17 ± 1.68  (2.28 – 8.06) | 7.43 ± 1.69  (4.78 – 10.33) |
| Sphere Ø 28 mm | 7.51 ± 2.10  (3.53 – 9.43) | 10.82 ± 1.97  (6.98 – 13.08) |
| Sphere Ø 37 mm | 10.55 ± 3.35  (4.49 – 14.19) | 15.79 ± 3.14  (10.08 – 19.22) |
| Lung insert Ø 50 mm (Ø 30 mm VOI) | -1.76 ± 0.40  (-2.20 – -1.02) | -2.69 ± 0.34  (-3.14 – -2.08) |

## Choice of reconstruction parameters

As stated in the main text was the choice of reconstruction parameters for the vendor-specific Siemens reconstructions based on the current clinical practice. The reconstruction parameters for the vendor-specific GE reconstructions were subsequently chosen to produce visually similar images with comparable noise levels as for the Siemens reconstructions. The convergence of the vendor-specific TEW reconstructions of the 15% wide main photopeak window are presented in Figure S.5. The number of iterations were varied between 1 to 10 for the vendor-specific GE reconstructions and between 1 to 15 iterations vendor-specific Siemens reconstructions. The number of subsets was fixed to 8 for all reconstructions. Data acquired with GE 870 and with Siemens Intevo 2 are used to represent each of the vendors investigated in this study. The values corresponding to the chosen reconstruction parameters are displayed in the shaded areas of each subplot; 4 iterations and 8 subsets for GE and 10 iterations and 8 subsets for Siemens.

The steep noise (COV) build-up in the vendor-specific GE reconstruction necessitated limiting of the number of iterations used in the reconstructions in order for this metric to be comparable to the vendor-specific Siemens reconstruction. Plausibly there is a small gain in the CRCs for vendor-specific GE reconstructions when iterating further, but, with the rapid increase of the noise, this comes at cost of reduced CNRs. For Siemens, the CRC of the largest sphere appears to be almost fully converged for the chosen reconstruction parameters.


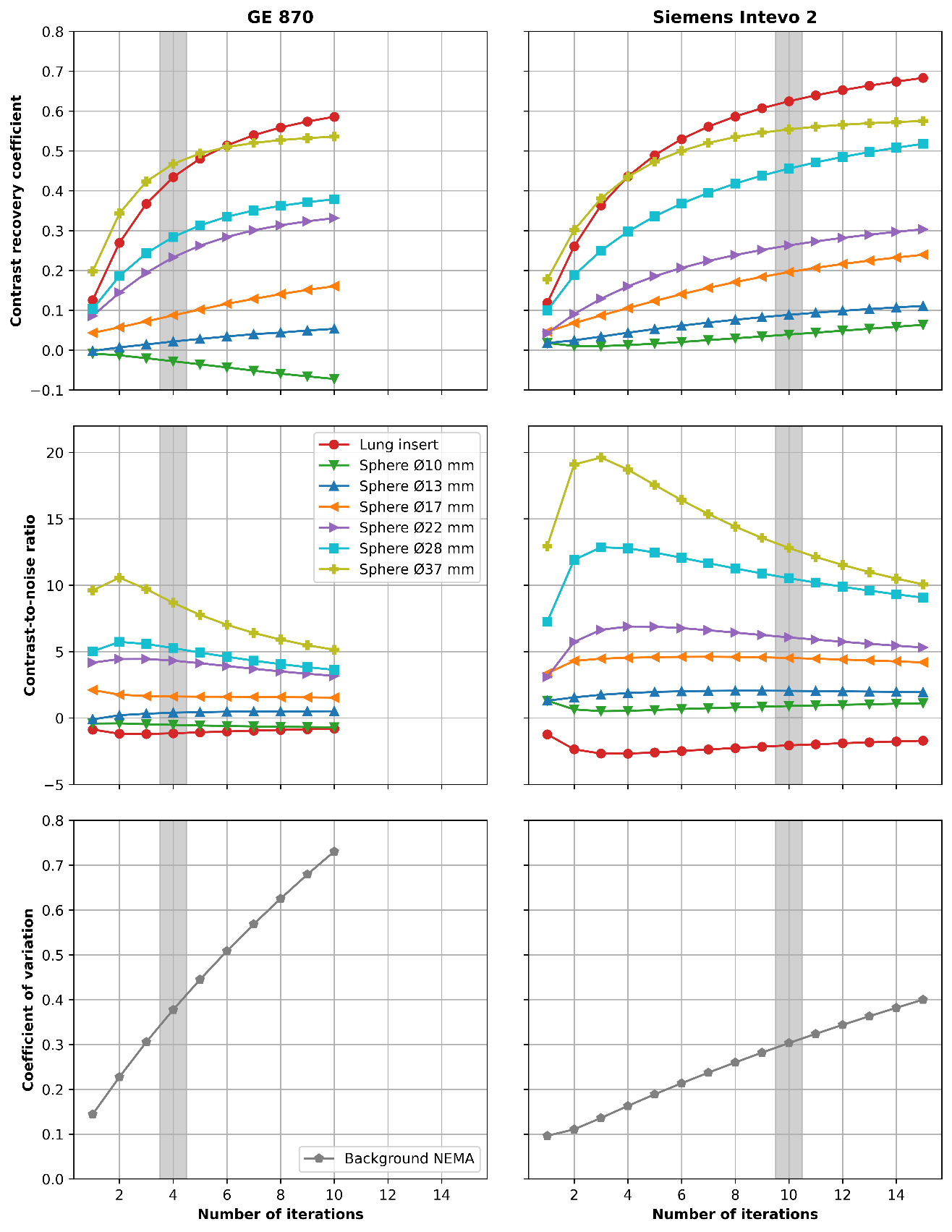


Figure S.5 Convergence of the TEW reconstruction for the data with a 15% wide main photopeak (presented in the main text). CRC, CNR, and COV as a function of number of iterations used in the reconstruction of the NEMA phantom for GE 870 (first column) and Siemens Intevo 2 (second column). The number of subsets was fixed to 8 for all reconstructions. The shaded areas represents the reconstruction parameters that were used for the GE- and the Siemens-specific reconstructions, respectively.

## Filtering of NEMA IEC phantom data

Post-reconstruction filtering of the SPECT images of the NEMA IEC phantom was carried out using an in-house developed script in Python. The contrast recovery and the contrast-to-noise ratio of the filtered images are presented below. Gaussian filters with 5, 10, or 15 mm full width half maximum (FWHM) were applied. The unfiltered data (also presented in the main text) is denoted by a filter of 0 mm FWHM.

In general, the contrast recovery is reduced with increasing filter size, meaning that there is a loss of detail in the SPECT images. On the other hand, the contrast-to-noise ratio increases with increasing filter size as a result of the lower background noise. Applying a Gaussian filter with a 10 to 15 mm FWHM to the GE data will result in a comparable CNR as for the unfiltered (0 mm FWHM) Siemens data. Note that applying this filter simultaneously results in a loss of CRC.

As previously noted; the vendor-specific reconstructions of the GE data with a main photopeak window of 15% were reconstructed utilizing 4 iterations and 8 subsets, while the GE data with a main photopeak window of 20% were reconstructed using 10 iterations and 8 subsets.

### Contrast recovery

CRCs of the filtered data of the NEMA IEC phantom is shown in Figure S.6 to Figure S.12 for each of the scanners in this study. The data presented in the main text is denoted by a filter of 0 mm FWHM.


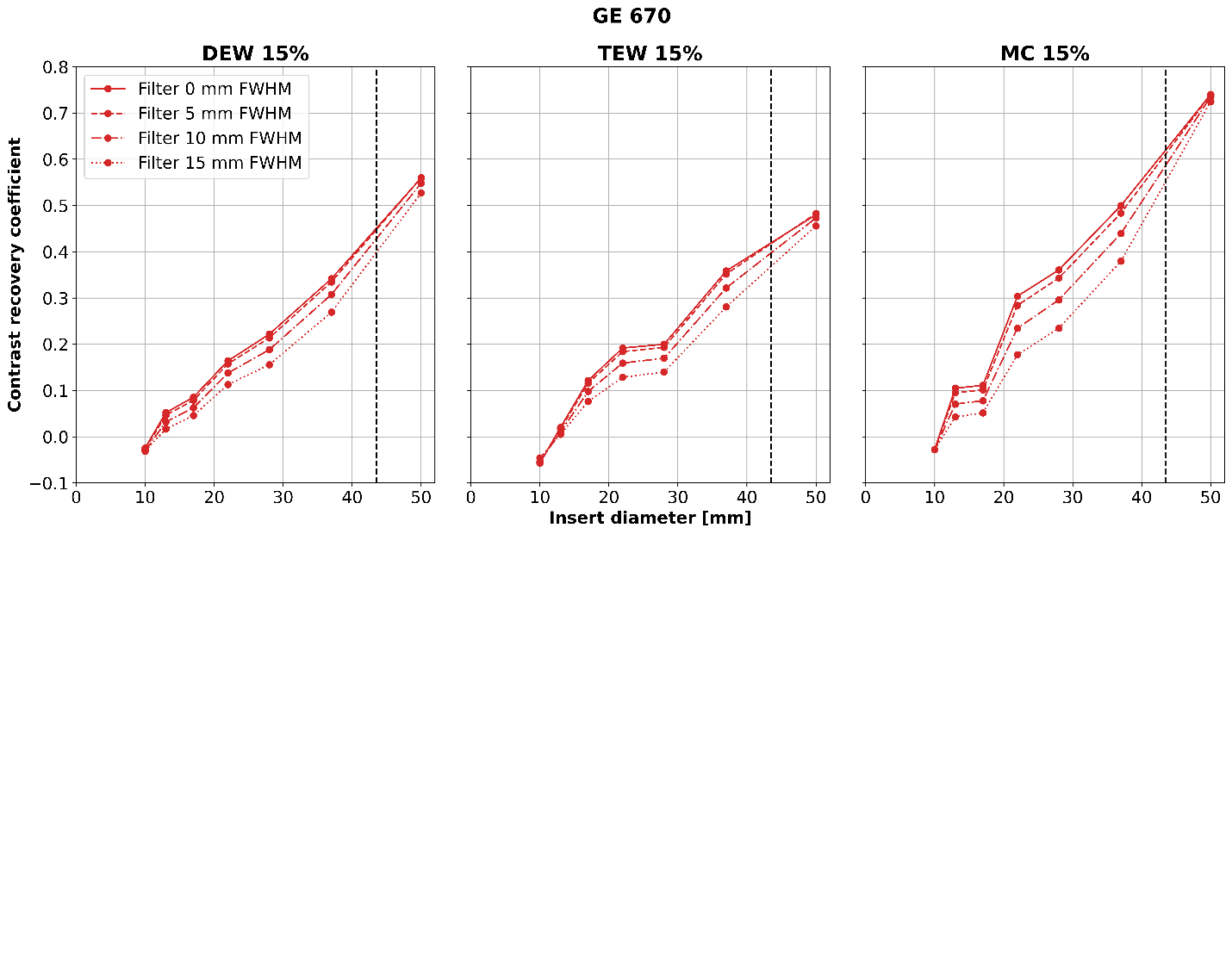


Figure S.6 GE 670: CRCs for different filter sizes as a function of the insert diameters of the NEMA IEC phantom. The vertical, black dashed line separates the markers for the spherical inserts (on the left) from the marker of the lung insert (on the right). The CRCs decrease with larger filter size


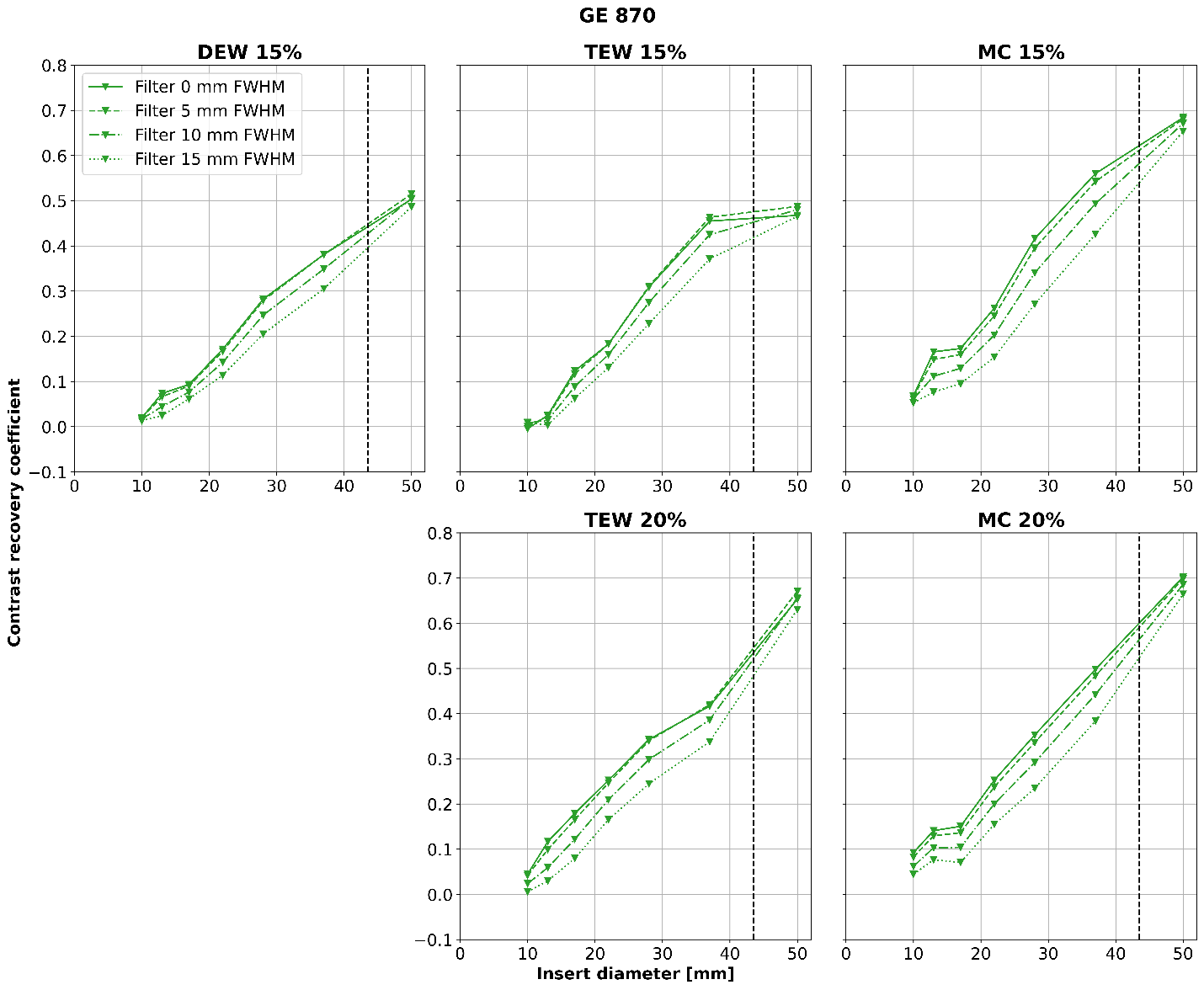


Figure S.7 GE 870: Mean CRC (averaged over the three consecutive acquisitions) for different filter sizes as a function of the insert diameters of the NEMA IEC phantom. The vertical, black dashed line separates the markers for the spherical inserts (on the left) from the marker of the lung insert (on the right). The CRCs generally decrease with larger filter size


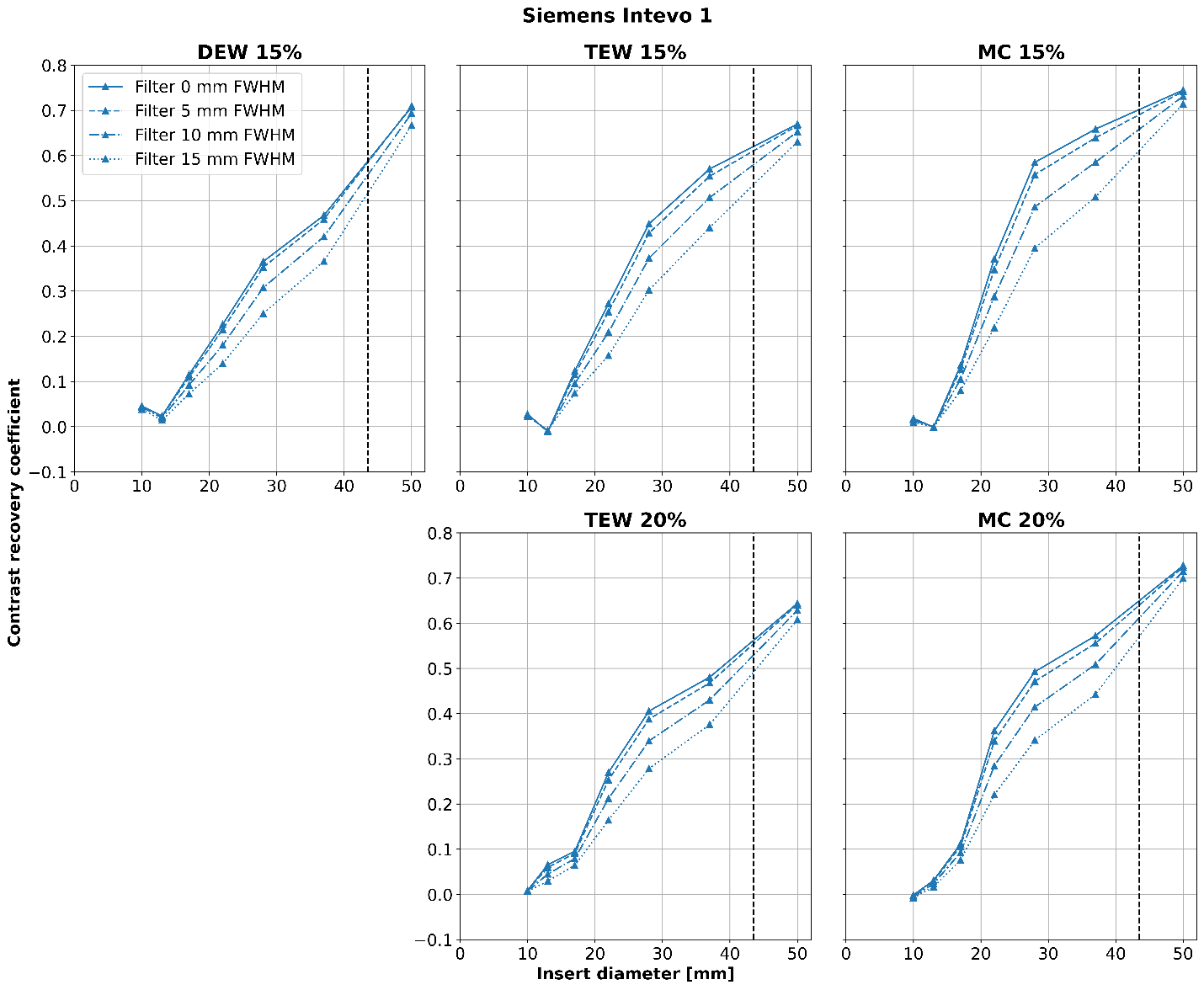


Figure S.8 Siemens Intevo 1: Mean CRC (averaged over the three consecutive acquisitions) for different filter sizes as a function of the insert diameters of the NEMA IEC phantom. The vertical, black dashed line separates the markers for the spherical inserts (on the left) from the marker of the lung insert (on the right). The CRCs generally decrease with larger filter size


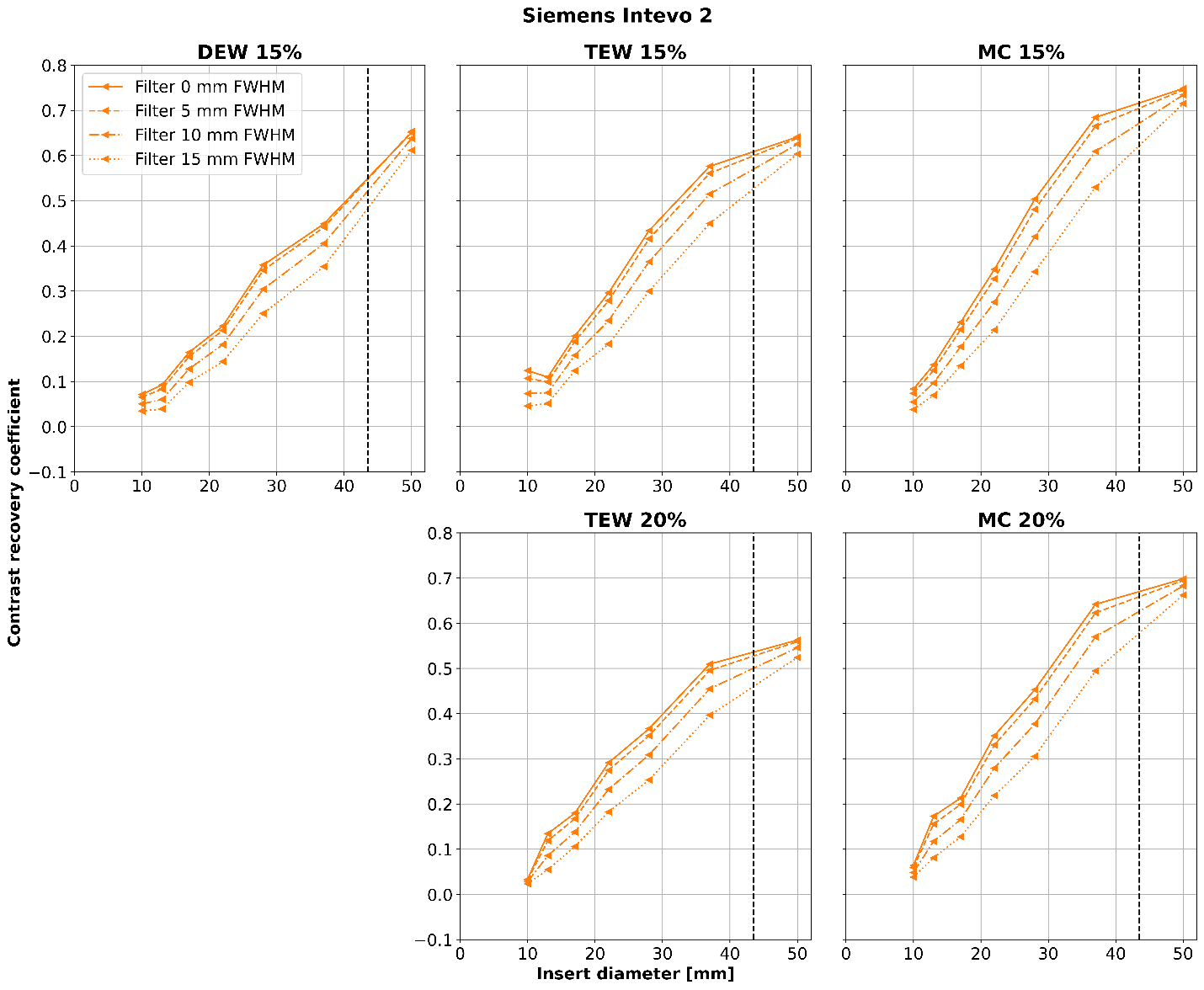


Figure S.9 Siemens Intevo 2: Mean CRC (averaged over the three consecutive acquisitions) for different filter sizes as a function of the insert diameters of the NEMA IEC phantom. The vertical, black dashed line separates the markers for the spherical inserts (on the left) from the marker of the lung insert (on the right). The CRCs decrease with larger filter size


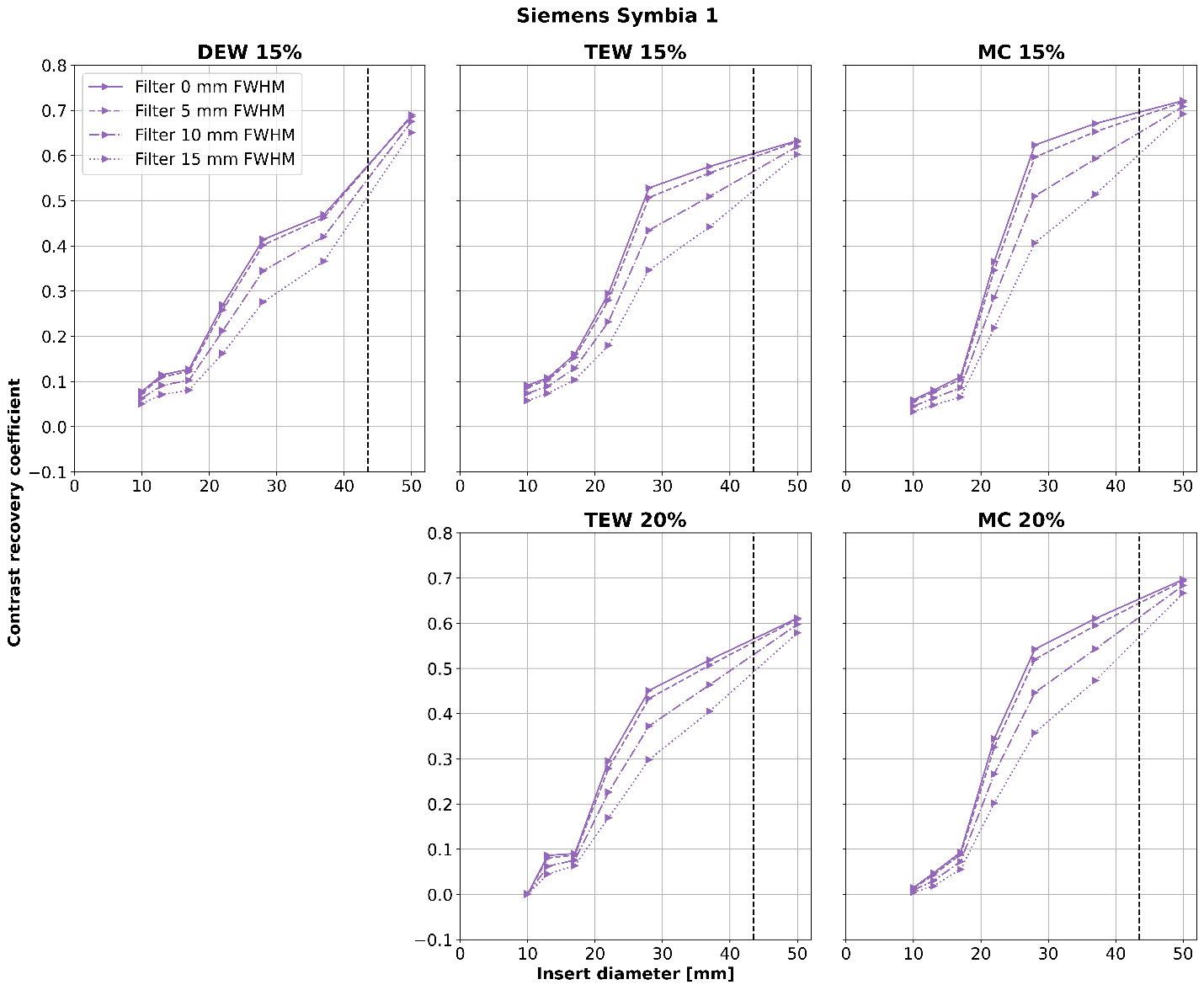


Figure S.10 Siemens Symbia 1: Mean CRC (averaged over the three consecutive acquisitions) for different filter sizes as a function of the insert diameters of the NEMA IEC phantom. The vertical, black dashed line separates the markers for the spherical inserts (on the left) from the marker of the lung insert (on the right). The CRCs decrease with larger filter size


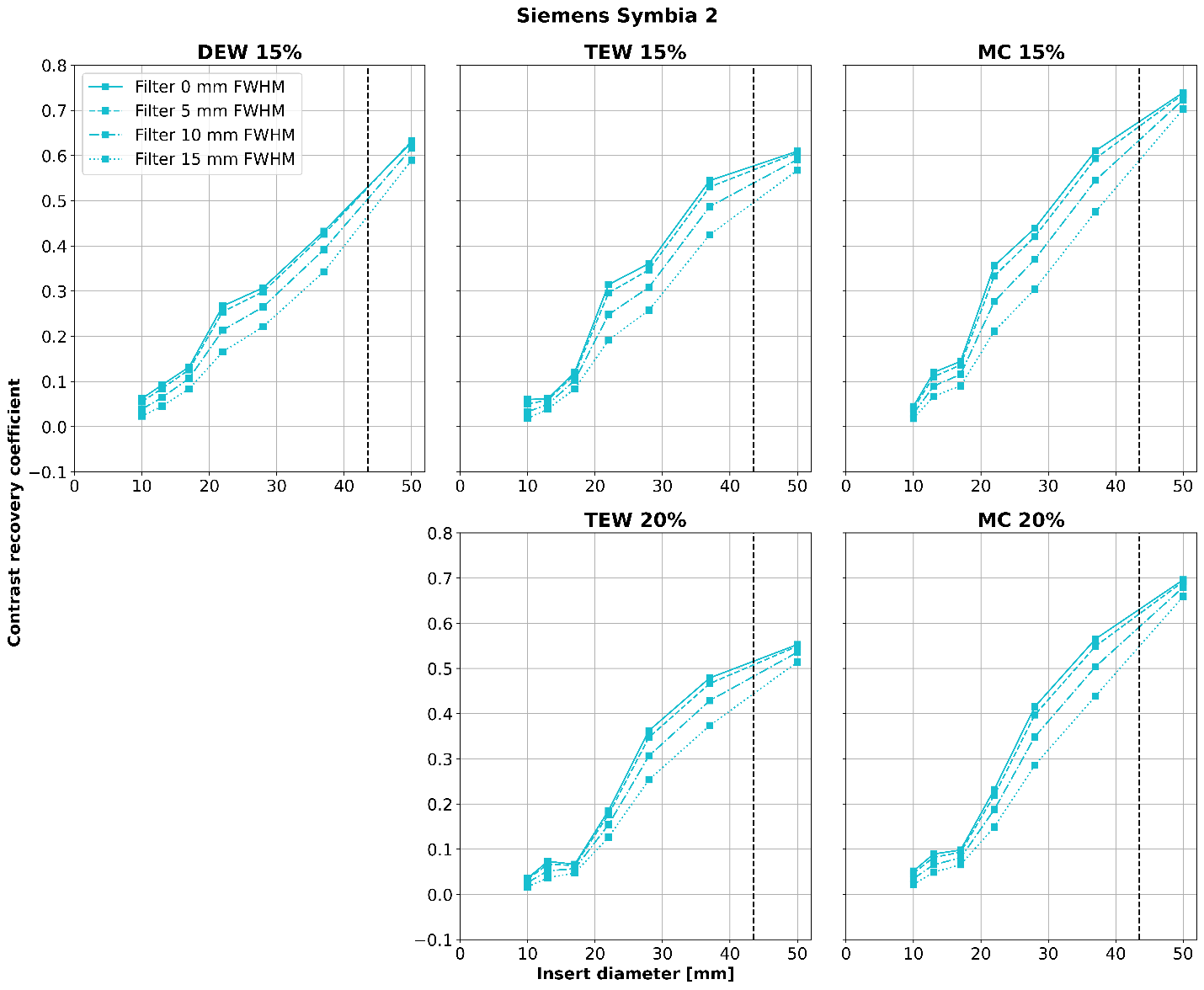


Figure S.11 Siemens Symbia 2: Mean CRC (averaged over the three consecutive acquisitions) for different filter sizes as a function of the insert diameters of the NEMA IEC phantom. The vertical, black dashed line separates the markers for the spherical inserts (on the left) from the marker of the lung insert (on the right). The CRCs decrease with larger filter size


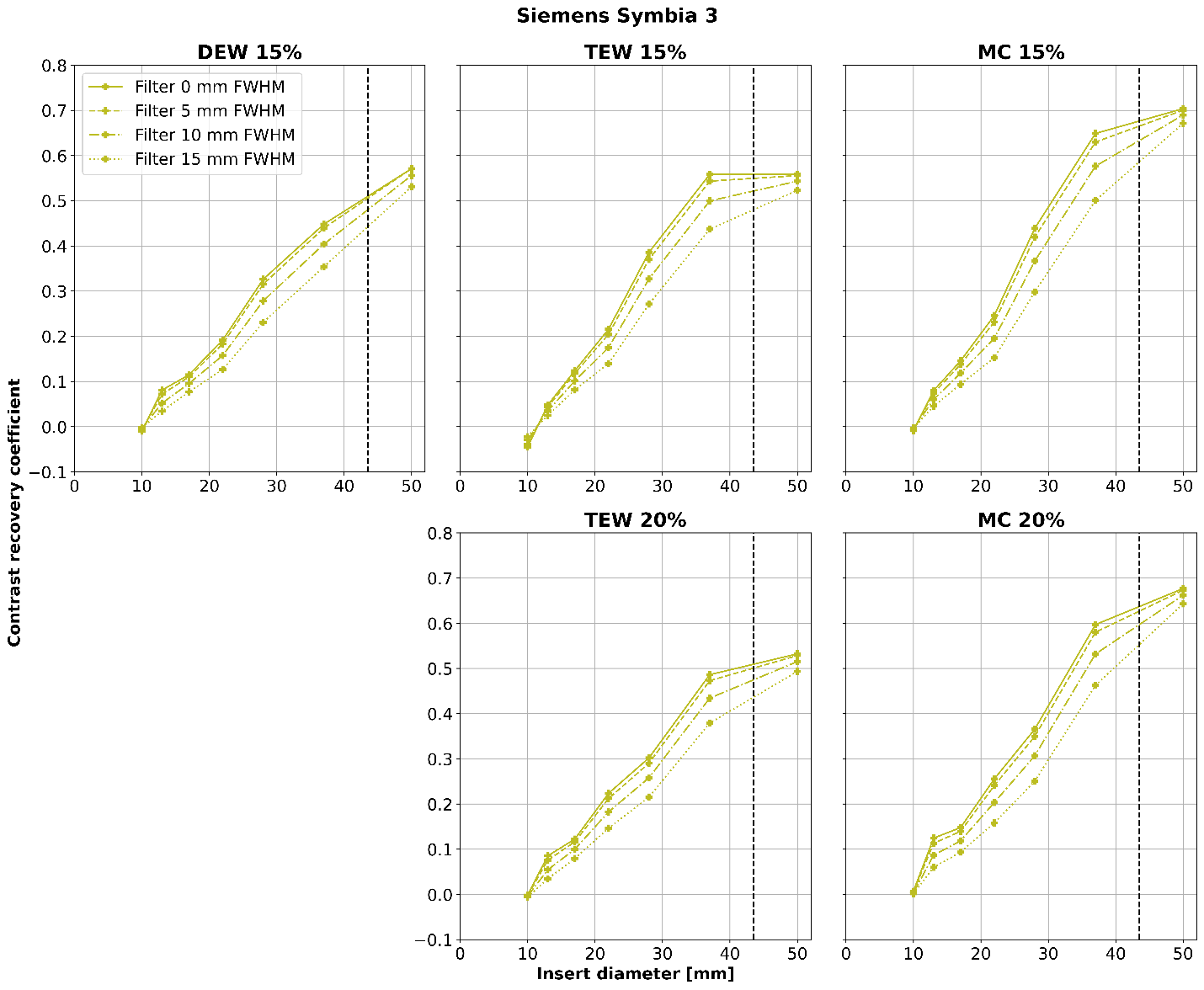


Figure S.12 Siemens Symbia 3: Mean CRC (averaged over the three consecutive acquisitions) for different filter sizes as a function of the insert diameters of the NEMA IEC phantom. The vertical, black dashed line separates the markers for the spherical inserts (on the left) from the marker of the lung insert (on the right). The CRCs decrease with larger filter size

### Contrast-to-noise ratio

CNRs of the filtered data of the NEMA IEC phantom is shown in Figure S.13 to Figure S.19 for each of the scanners in this study. The data presented in the main text is denoted by a filter of 0 mm FWHM.


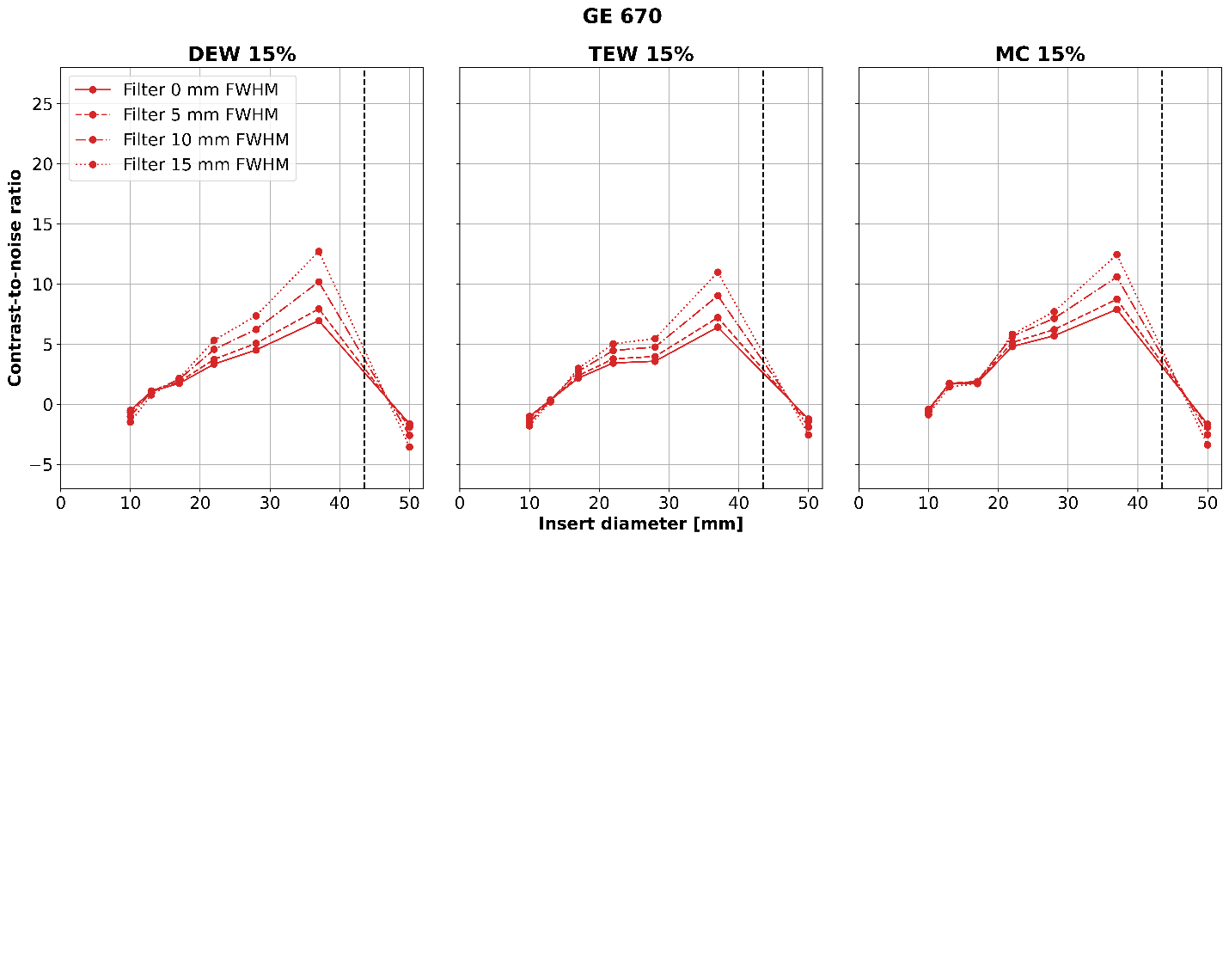


Figure S.13 GE 670: CNR for different filter sizes as a function of the insert diameters of the NEMA IEC phantom. The vertical, black dashed line separates the markers for the spherical inserts (on the left) from the marker of the lung insert (on the right). The absolute value of the CNRs generally increase with larger filter size


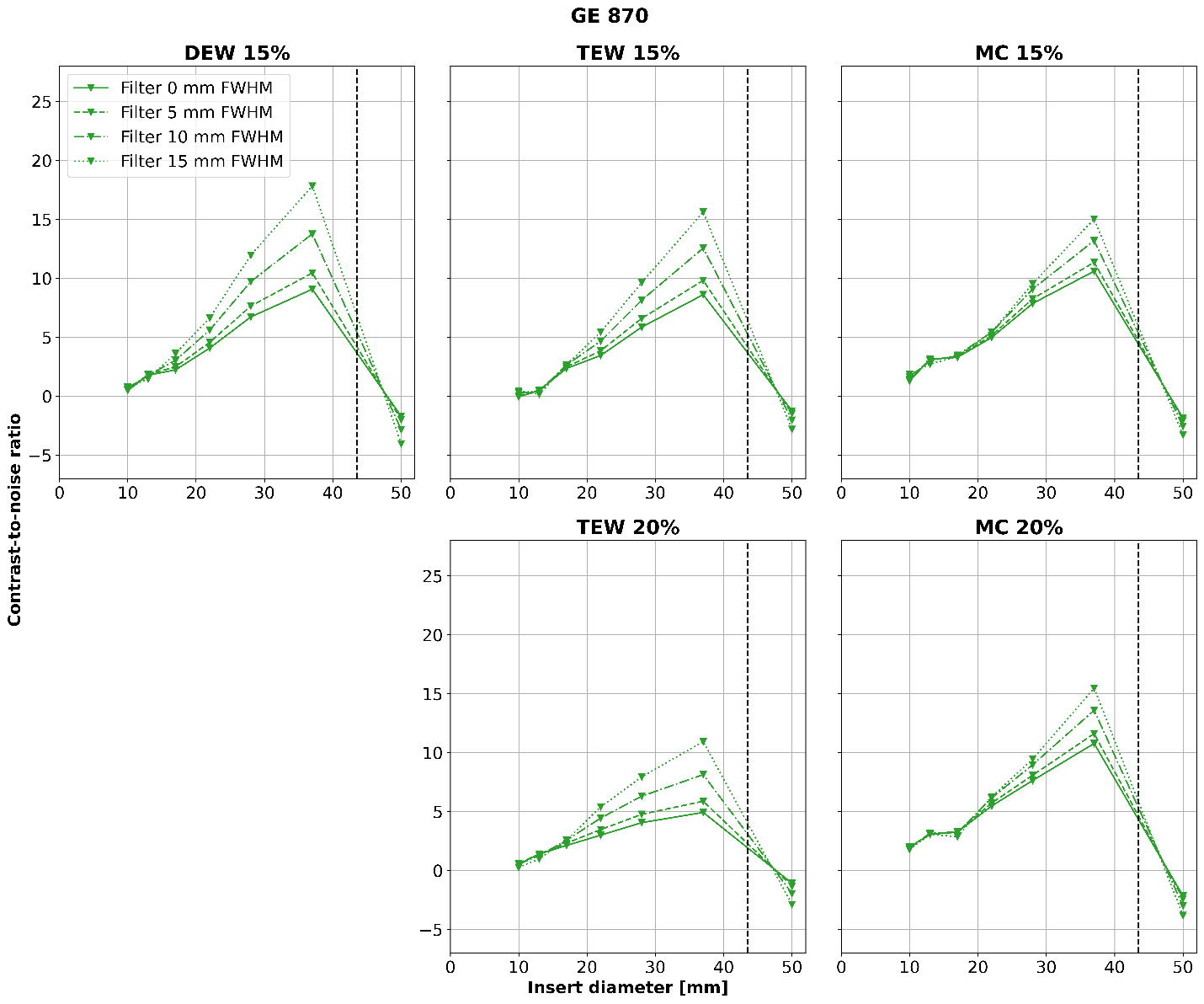


Figure S.14 GE 870: Mean CNR (averaged over the three consecutive acquisitions) for different filter sizes as a function of the insert diameters of the NEMA IEC phantom. The vertical, black dashed line separates the markers for the spherical inserts (on the left) from the marker of the lung insert (on the right). The absolute value of the CNRs generally increase with larger filter size


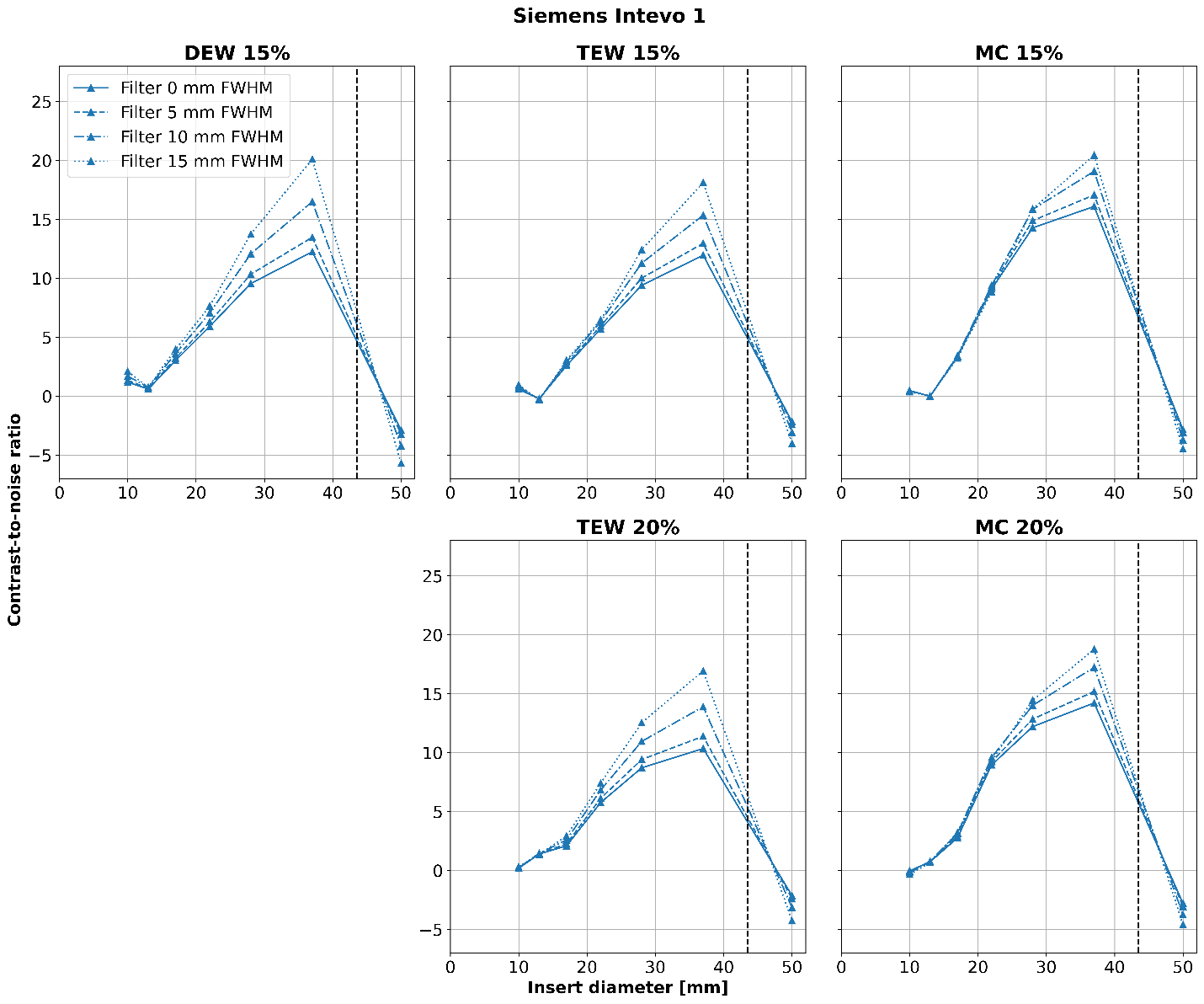


Figure S.15 Siemens Intevo 1: Mean CNR (averaged over the three consecutive acquisitions) for different filter sizes as a function of the insert diameters of the NEMA IEC phantom. The vertical, black dashed line separates the markers for the spherical inserts (on the left) from the marker of the lung insert (on the right). The absolute value of the CNRs generally increase with larger filter size


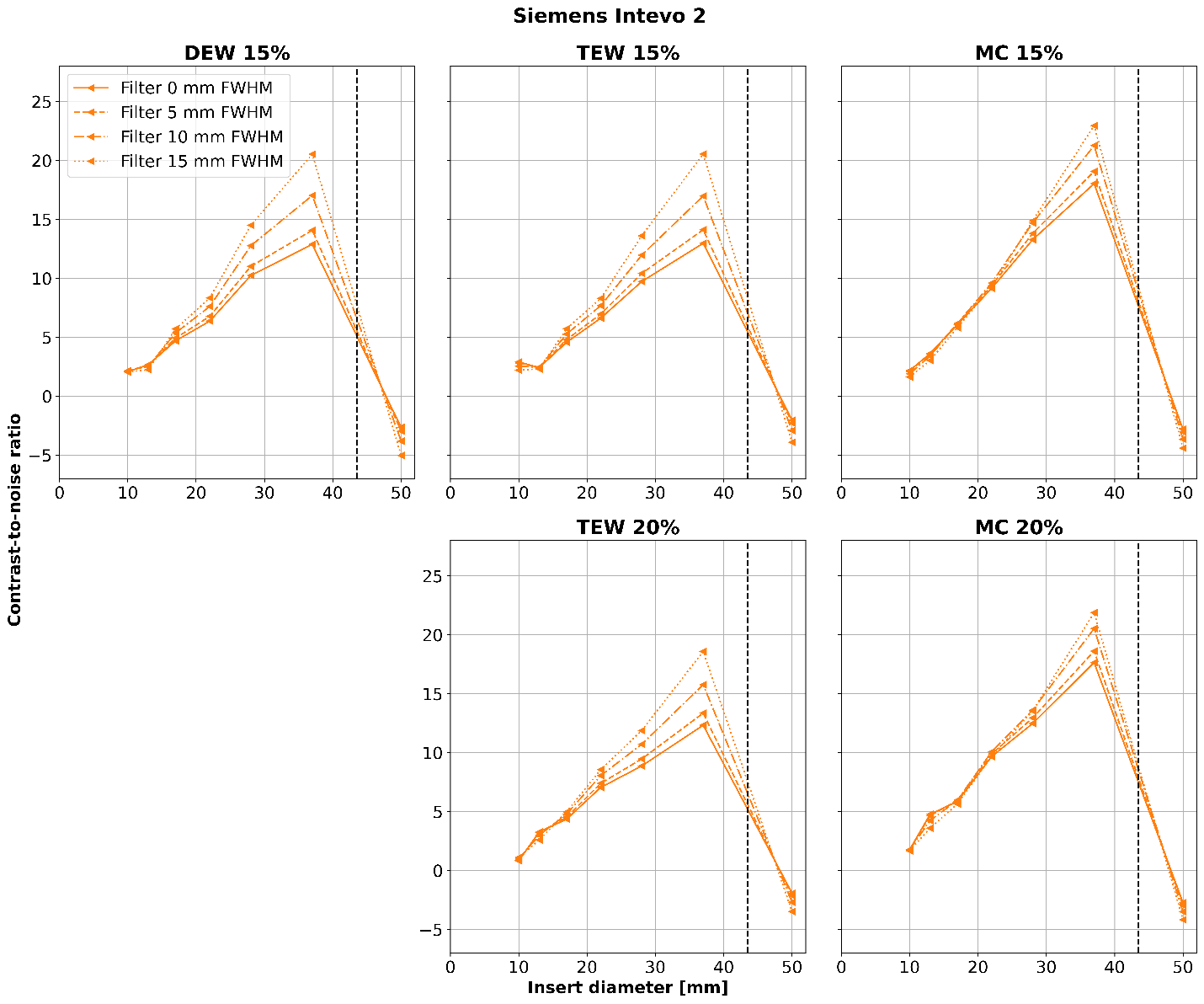


Figure S.16 Siemens Intevo 2: Mean CNR (averaged over the three consecutive acquisitions) for different filter sizes as a function of the insert diameters of the NEMA IEC phantom. The vertical, black dashed line separates the markers for the spherical inserts (on the left) from the marker of the lung insert (on the right). The absolute value of the CNRs generally increase with larger filter size


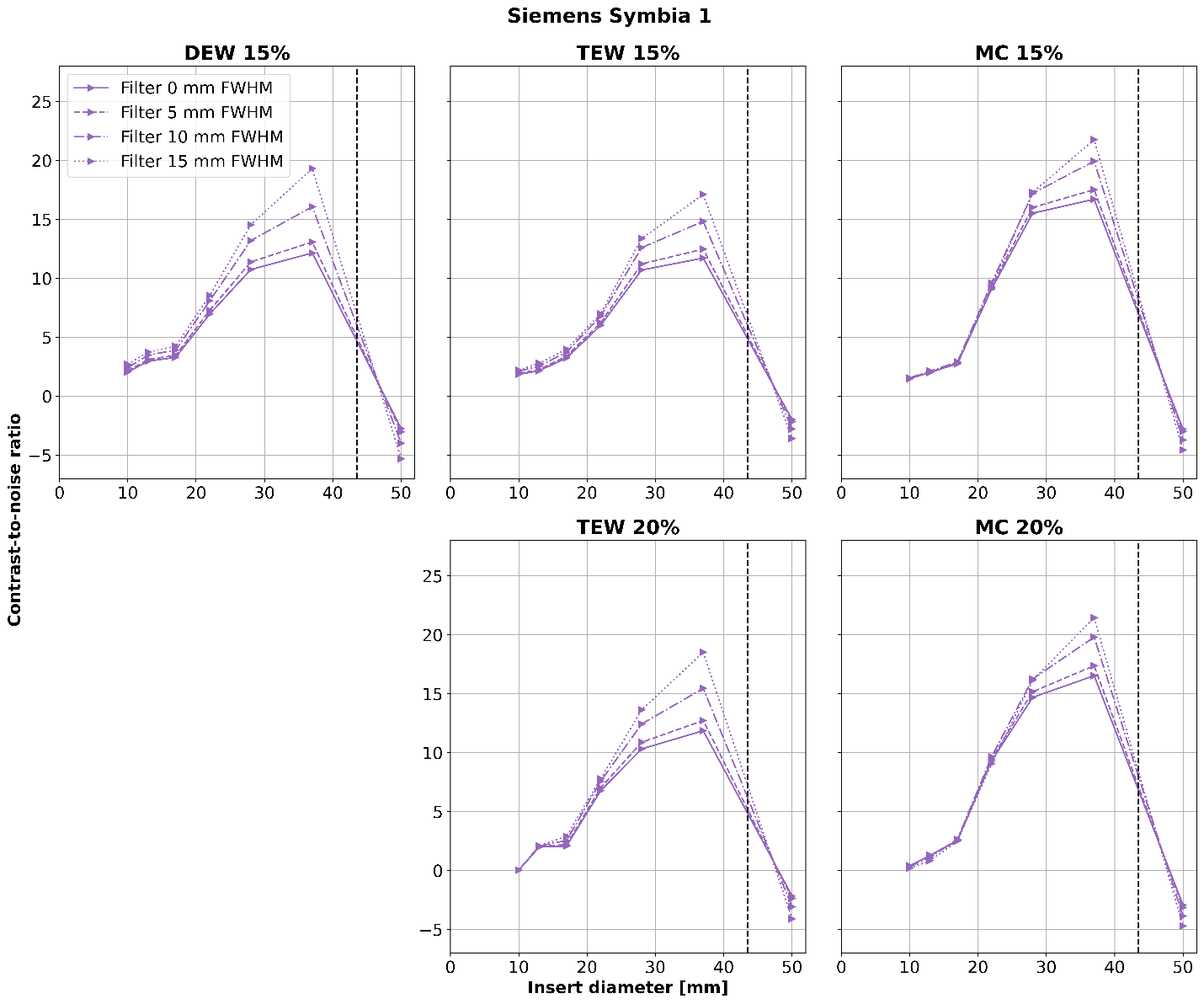


Figure S.17 Siemens Symbia 1: Mean CNR (averaged over the three consecutive acquisitions) for different filter sizes as a function of the insert diameters of the NEMA IEC phantom. The vertical, black dashed line separates the markers for the spherical inserts (on the left) from the marker of the lung insert (on the right). The absolute value of the CNRs generally increase with larger filter size


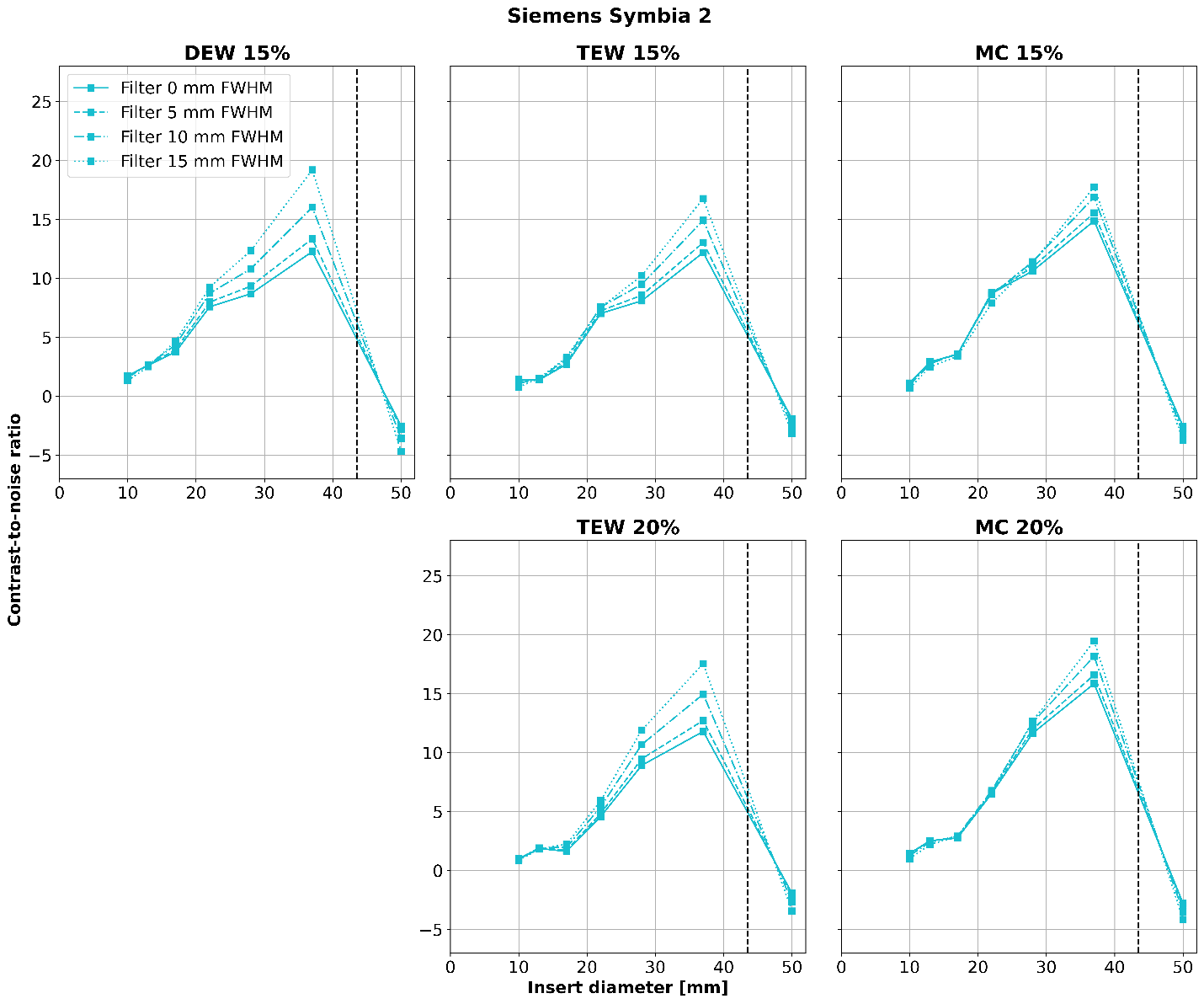


Figure S.18 Siemens Symbia 2: Mean CNR (averaged over the three consecutive acquisitions) for different filter sizes as a function of the insert diameters of the NEMA IEC phantom. The vertical, black dashed line separates the markers for the spherical inserts (on the left) from the marker of the lung insert (on the right). The absolute value of the CNRs generally increase with larger filter size


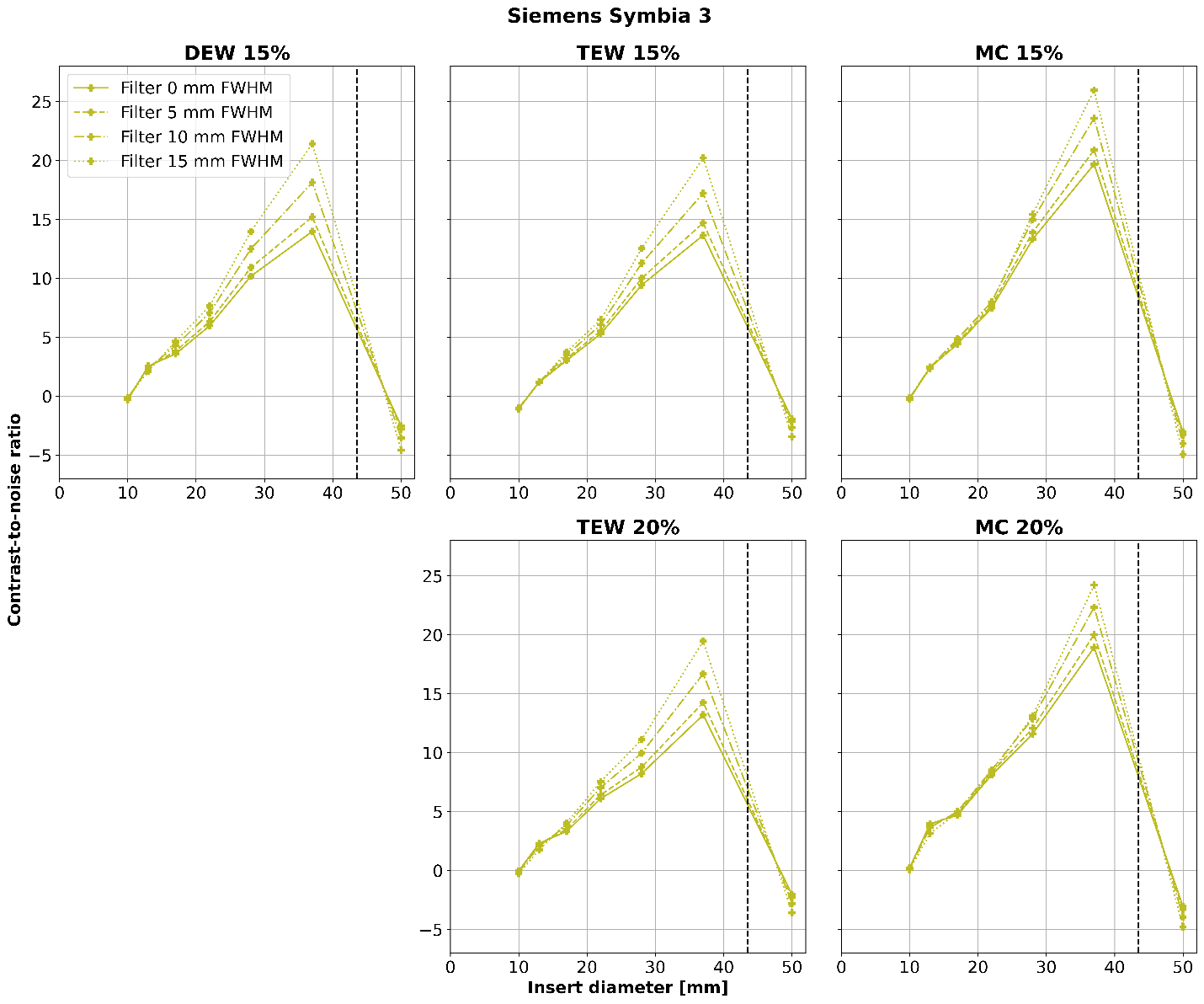


Figure S.19 Siemens Symbia 3: Mean CNR (averaged over the three consecutive acquisitions) for different filter sizes as a function of the insert diameters of the NEMA IEC phantom. The vertical, black dashed line separates the markers for the spherical inserts (on the left) from the marker of the lung insert (on the right). The absolute value of the CNRs generally increase with larger filter size
